# Neuroglobin plays as tumor suppressor by disrupting the stability of GPR35 in colorectal cancer

**DOI:** 10.1186/s13148-023-01472-2

**Published:** 2023-04-01

**Authors:** Qin Xiang, Dishu Zhou, Xinni Xiang, Xin Le, Chaoqun Deng, Ran Sun, Chunhong Li, Huayang Pang, Jin He, Zeze Zheng, Jun Tang, Weiyan Peng, Xi Peng, Xiaoqian He, Fan Wu, Jingfu Qiu, Yongzhu Xu, Tingxiu Xiang

**Affiliations:** 1 Department of Oncology, The First Affiliated Hospital of Chongqing Medical University, Chongqing, 400016 China; 2 Chongqing Key Laboratory of Translational Research for Cancer Metastasis and Individualized Treatment, Chongqing University Cancer Hospital, Chongqing, 400030 China; 3 School of Public Health and Management, Chongqing Medical University, Chongqing, 400016 China; 4 Chongqing Blood Center, Chongqing, 400015 China; 5 West China School of Medicine, Sichuan University, Chengdu, 610065 Sichuan China

**Keywords:** NGB, GPR35, Biomarker, Methylation, Tumor angiogenesis

## Abstract

**Background:**

The incidence of colorectal cancer (CRC) has increased in recent years. Identification of accurate tumor markers has become the focus of CRC research. Early and frequent DNA methylation tends to occur in cancer. Thus, identifying accurate methylation biomarkers would improve the efficacy of CRC treatment. Neuroglobin (NGB) is involved in neurological and oncological diseases. However, there are currently no reports on epigenetic regulation involvement of NGB in CRC.

**Results:**

NGB was downregulated or silenced in majority CRC tissues and cell lines. The hypermethylation of NGB was detected in tumor tissue, but no or a very low methylation frequency in normal tissues. Overexpression of NGB induced G2/M phase arrest and apoptosis, suppressed proliferation, migration, invasion in vitro, and inhibited CRC tumor growth and angiogenesis in vivo. Isobaric tag for relative and absolute quantitation (Itraq)-based proteomics identified approximately 40% proteins related to cell–cell adhesion, invasion, and tumor vessel formation in the tumor microenvironment, among which GPR35 was proved critical for NGB-regulated tumor angiogenesis suppression in CRC.

**Conclusions:**

NGB, an epigenetically silenced factor, inhibits metastasis through the GPR35 in CRC. It is expected to grow into a potential cancer risk assessment factor and a valuable biomarker for early diagnosis and prognosis assessment of CRC.

**Supplementary Information:**

The online version contains supplementary material available at 10.1186/s13148-023-01472-2.

## Background

Colorectal cancer (CRC) is a digestive tract tumor with a high degree of malignancy, and its incidence has been increasing in recent years [1]. Most CRC patients are not eligible for surgery due to late diagnosis. New targeted drugs and immune therapy agents have been developed from CRC markers and associated signaling pathways, and novel therapeutic strategies have achieved encouraging results [2–4]. However, high tumor heterogeneity and challenges form lacking early detection of CRC limited the efficacy of CRC treatments [5, 6]. Early and frequent DNA methylation occurs in cancer. Changes in DNA methylation in cancer have been heralded as promising targets for the development of powerful diagnostic, prognostic, and predictive biomarkers [2, 7]. Identification of accurate methylation biomarkers would improve the efficacy of CRC treatment.

Several methylation biomarkers related to prognosis and prediction of cancer were identified by our team, such as *ZDHHC1*, *OPCML*, *ADAMTS9*, *PLCD1* [8–10]. In our previous study, we demonstrated that promoter hypermethylation contributes to cytoglobin (*CYGB*, the fifth member of globin family) suppression in breast cancer [11]. Neuroglobin (*NGB*), another member of globin family, was originally discovered in the brains of humans and mice in 2000 [12]. The role of NGB in neurological diseases and nervous system tumors has been reported extensively [13, 14]. However, its function in non-neurological tumors, such as breast cancer [15], liver cancer [16] and lung cancer [17], remains to explore detailedly. There are currently no reports on epigenetic regulation involvement of *NGB* in CRC. The objectives of this study were to determine whether promoter methylation of *NGB* affects CRC progression and to elucidate the underlying mechanisms.

In the present study, we showed that *NGB* is downregulated due to promoter methylation in CRC tissues, and ectopic *NGB* expression inhibited cell proliferation, suppressed cell apoptosis, and caused cell cycle arrest in CRC cell lines. In a nude mouse xenograft tumor model, NGB delayed tumor growth and suppressed intratumoral vascular endothelial cell infiltration. Vascular endothelial growth factor (VEGF), an essential growth factor for vascular endothelial cells, was well defined contributing to tumor angiogenesis [18]. The union of antineoplastic and antiangiogenic drugs is a common treatment in metastatic CRC. Recent research reported that activation of the GPR35 pathway drives angiogenesis in the tumor microenvironment [19]. Our data showed that the tumor-suppressive function of NGB was mediated by the inhibition of the GPR35/angiogenesis axis. Taken together, the current findings suggest that NGB may act not only as a new predictive biomarker but also an effective marker for risk assessment of CRC metastasis.

## Results

### NGB is downregulated due to promoter methylation in colorectal cancer and associated with cancer metastasis

**Fig. 1:**
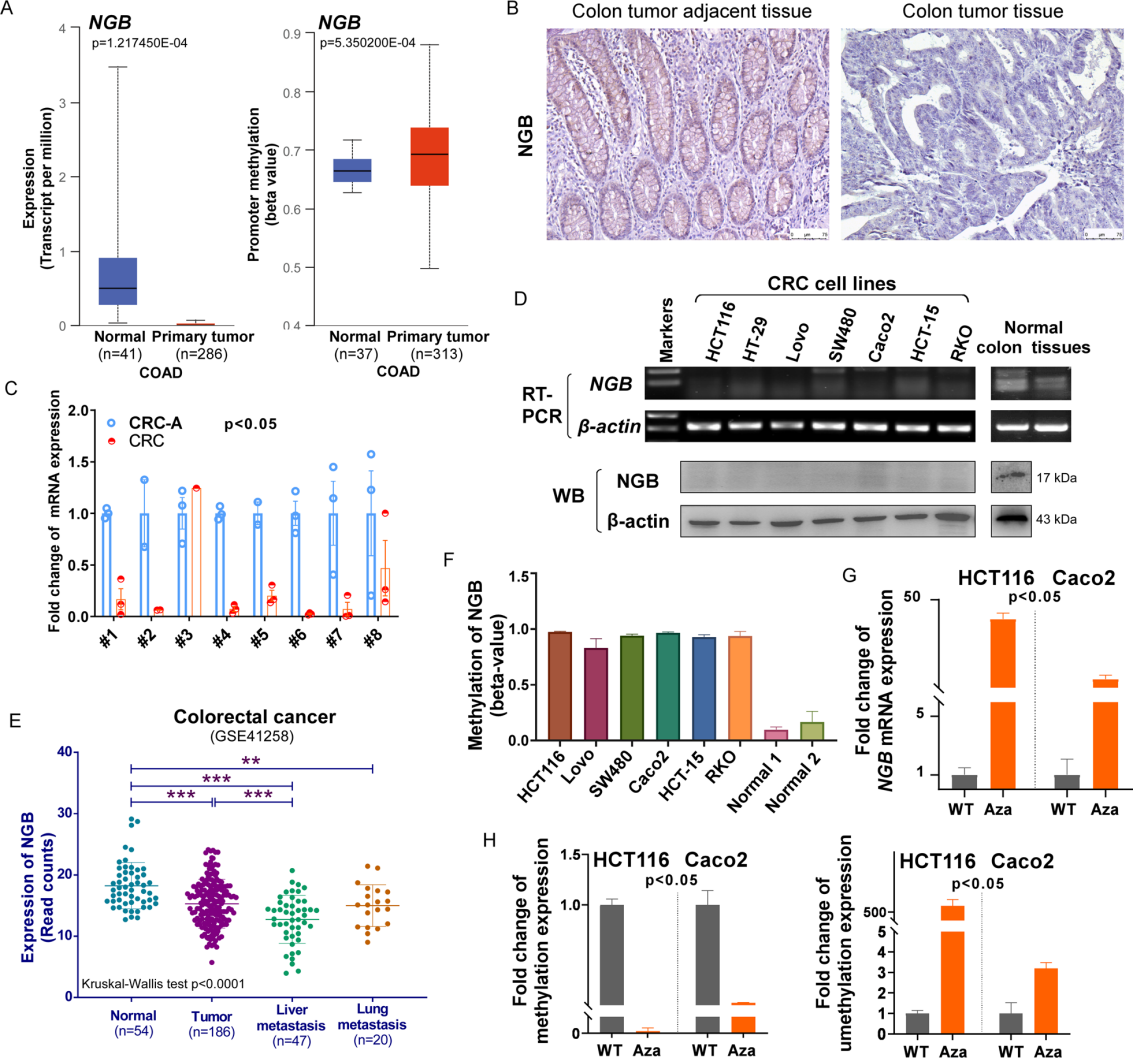
NGB is downregulated due to promoter methylation in CRC **A** The mRNA expression and promoter methylation of NGB in COAD. COAD: colon adenocarcinoma, data from UALACN (http://ualcan.path.uab.edu/). **B** Protein level of NGB in paired human colorectal carcinoma tissues detected by IHC staining, *N* = 3. **C** Fold-change of *NGB* mRNA expression detected by qRT-PCR in CRC and CRC-A. CRC: CRC, CRC-A: CRC-adjacent. **D** RT-PCR and Western blot results showing mRNA expression of *NGB* in CRC cell lines. **E** Expression of NGB in normal colon, primary tumor, liver metastasis and lung metastasis of CRC, data from GSE412584. **F** Methylated analysis of NGB in CRC cell lines, *p* < 0.05. **G** The mRNA expression level of *NGB* detected by qRT-PCR after treatment with demethylating agent Aza. **H** Methylation status of NGB detected by qMSP after treatment with demethylating agent Aza. All experiments were performed in triplicate. *p < 0.05, **p < 0.01, ***p < 0.001

**Fig. 2:**
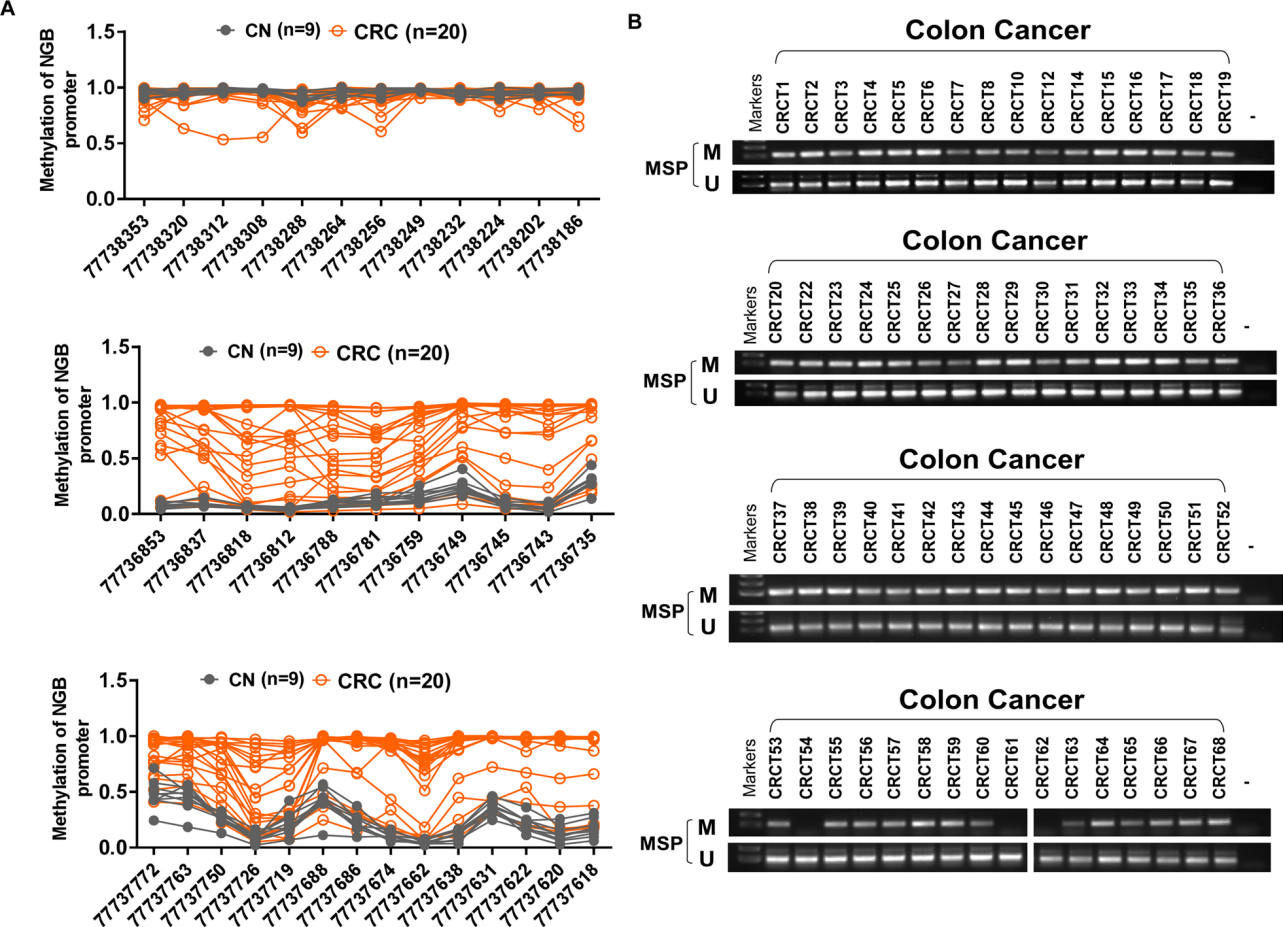
Methylated status of NGB in CRC tissues. **A** Methylation of NGB promoter including 37 methylation sites detected by Methyltarget; **B** The methylated status of NGB in CRC detected by MSP, *N* = 64

A search of The University of ALabama at Birmingham CANcer data analysis Portal [20, 21] (UALCAN, http://ualcan.path.uab.edu/) showed that the expression of NGB is lower in CRC samples than in adjacent tissues (Fig. 1A left, Additional file 1: Fig. S1A), while the methylation status of NGB is higher (Fig. 1A right). Immunohistochemistry (IHC) staining showed that NGB protein levels were lower in colon tumor tissues than in normal colon tissues (Fig. 1B); Human Protein Atlas (HPA) database also showed similar tendency, with almost half exhibiting weak/negative expression in total 11 CRC tissues (45.5%), and nearly all showing moderate expression in normal colon (Additional file 1: Fig. S2, image available from version 22.0.proteinatlas.org) [22, 23]. *NGB* expression in eight paired clinical tissues was detected by qRT-PCR, and the results showed that NGB was lowly expressed in tumor 7 out of 8 pairs (87.5%) (Fig. 1C). Assessment of *NGB* expression by RT-PCR and Western blot in CRC cell lines and normal colon tissues showed that *NGB* was downregulated or silenced in cancer (Fig. 1D). NGB expression level was low among CRC patients, especially those with liver metastasis (Fig. 1E, data from GSE41258). The expression level of NGB has been reported not significant on the survival of CRC patients [24], whereas high expression of NGB might potentially protect them from relapse (Additional file 1: Fig. S1B). Moreover, we discovered hypermethylation status of CRC cell lines compared with normal colon cells (Fig. 1F). The mRNA expression of NGB can be restored after treatment with demethylation agent 5-aza-2’-deoxycytidine (Aza) (Fig. 1G). Methylation expression of NGB promoter was significantly decreased after treatment with Aza (Fig. 1H left), as opposed to the unmethylation expression (Fig. 1H right). Methylation site of NGB promoter from 77,736,853 to 77,736,735 performed by Methyltarget, could ability to distinguish between tumor and adjacent (Fig. 2A). The methylated rate of NGB was 95.3% (61/64), detected by MSP (methylation special PCR) in CRC tissues (Fig. 2B). These results suggested that *NGB* is a frequently downregulated gene due to hypermethylation of promoter and negatively associated with liver metastasis in CRC.

### NGB suppresses CRC progression and inhibits tumor growth by affecting tumor inflammation

**Fig. 3:**
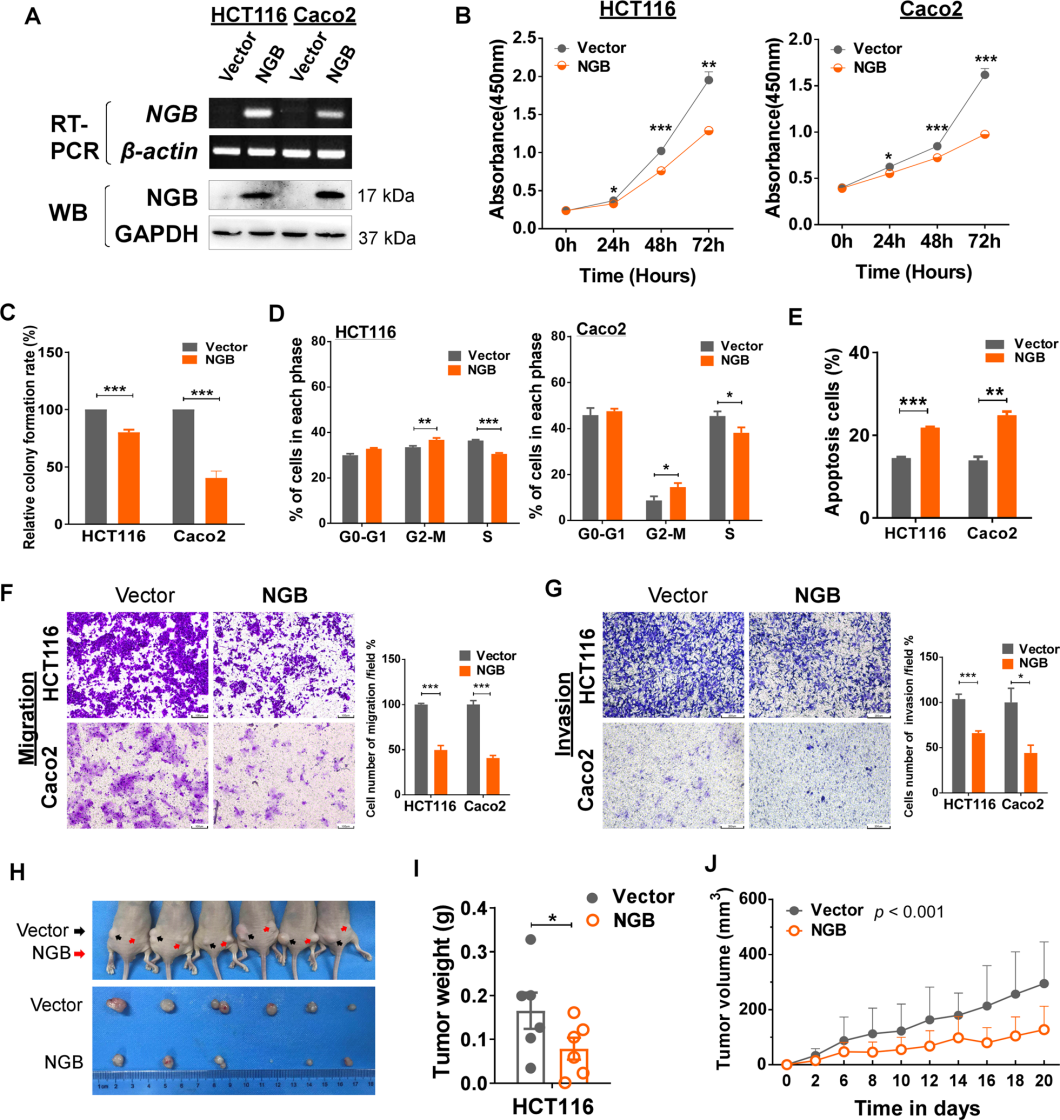
NGB inhibits cancer cell proliferation and inhibits tumor growth in vivo and in vitro. **A** Confirmation of NGB-OE determined by RT-PCR and Western blot (WB). **B** Cell viability analysis by CCK8 assay. **C** Analysis of cell proliferation by colony formation assay. **D** Percentage of in NGB or vector cells distributed in each phase. **E** Apoptotic ratio in NGB or Vector CRC cells. **F** Migration of CRC cells with or without NGB-OE; the statistical analysis is shown on the right. **G** Invasion of CRC cells with or without NGB-OE; the statistical analysis is shown on the right. **H** Photographs of subcutaneous tumors of nude mice taken after 20 days, *N* =  6. **I** The tumor weight was recorded. **J** The subcutaneous tumor volume of nude mice was measured every two days before the tumor volume exceeded 1 cm.^3^. *p < 0.05, **p < 0.01, ***p < 0.001

Vector- and *NGB-* stably expressing cell lines were constructed in HCT116 and Caco2 cells. The overexpression of NGB was confirmed by RT-PCR and Western blot (Fig. 3A). Cell proliferation was examined using CCK8 and colony formation assays, which indicated that NGB overexpression (NGB-OE) significantly inhibited the proliferation of HCT116 and Caco2 cells (*p* < 0.05, Fig. 3B) and resulted in fewer and smaller colonies than those in the control groups (*p* < 0.001, Fig. 3C, additional file 1: Fig. S3A).

Flow cytometric analysis was performed to investigate the anti-tumor effects of *NGB* on cell cycle progression and apoptosis. *NGB*-OE caused accumulation of cells in the G2/M phase compared with the control group (37.69% vs. 33.62% in HCT116; 12.6% vs. 6.82% in Caco2, *p* < 0.05, Fig. 3D, Additional file 1: Fig. S3B). Annexin V-FITC/PI staining was used to evaluate the effect of NGB on cell apoptosis in CRC. As shown in Fig. 3E and Additional file 1: Fig. S3C, the percentage of Annexin V PI-positive cells was higher in Caco2 and HCT116 cells overexpressing NGB than in the controls (7% increase in HCT116; 14.5% increase in Caco2, *p* < 0.01). These results indicated that *NGB* inhibits the proliferation of CRC cells by causing cell cycle arrest at G2/M phase and inducing cell apoptosis.

The effect of NGB on tumor cell metastasis was assessed using Transwell assay. Results showed that the number of cells passing through the membrane was less in *NGB*-OE than in control cells (Fig. 3F, G). Proteins regulating cell–cell adhesions were evaluated by Western blot; data showed that E-cadherin was increased, N-cadherin and Vimentin were decreased in NGB-OE than in control cells (Additional file 1: Fig. S3D). These data demonstrated that NGB suppresses migration and invasion of CRC cells.

The in vivo tumorigenic ability of NGB was subsequently examined on nude mice. HCT116 cells with or without NGB overexpression were implanted subcutaneously on both flanks, respectively. After 20 days of observation, xenograft tumors were excised from nude mice for further analysis. Tumor growth was markedly retarded, and tumor volume and weight were confined in the NGB-OE group (*p* < 0.05, Fig. 3H–J). H&E staining indicated that solid nests quantities in vector group were more than that in NGB-OE group (Additional file 1: Fig. S4A). At high magnification, diffuse inflammatory cell infiltration was higher and nuclear size was greater in the vector group than in the NGB-OE group, and the mean gray value of H&E was lower in the NGB-OE group than in the vector group (Additional file 1: Fig. S4B). In addition, IHC showed a distinct decrease of Ki67 (proliferation marker), microvessel density (MVD, the counts of positive staining of CD31, an endothelial vascular cell marker) and CD8α (cell infiltration marker) caused by ectopic NGB expression (Additional file 1: Fig. S4C, D). These data suggested that NGB suppresses tumorigenesis and tumor growth in nude mice xenografts possibly by inhibiting inflammatory cell infiltration and tumor endothelial formation.

### Overexpression of NGB inhibits tumor angiogenesis in CRC cells

We further examined the alterations occurring in response to NGB-OE and identified 278 differentially expressed proteins (DEPs) (fold change > 1.2, Qvalue < 0.05; 176 upregulated, 102 downregulated) by iTARQ analysis (Additional file 1: Fig. S5A). Analysis of DEPs by gene ontology (GO) showed that the enriched molecular functions of NGB were binding activity and catalytic activity (Additional file 1: Fig. S5B). Kyoto encyclopedia of genes and genomes (KEGG) annotation indicated that DEPs regulated several pathways related to environmental information processing (e.g., signal transduction, signaling molecules and interactions), human diseases (cancers, infectious), and organismal systems (immune, endocrine, digestive) (Additional file 1: Fig. S5C). Many DEPs were related to cellular processes and signaling such as signal transduction mechanisms, post-translational modifications, cell cycle control, and cytoskeleton, as determined in eukaryotic orthologous group (KOG) annotation (Additional file 1: Fig. S5D).

**Fig. 4:**
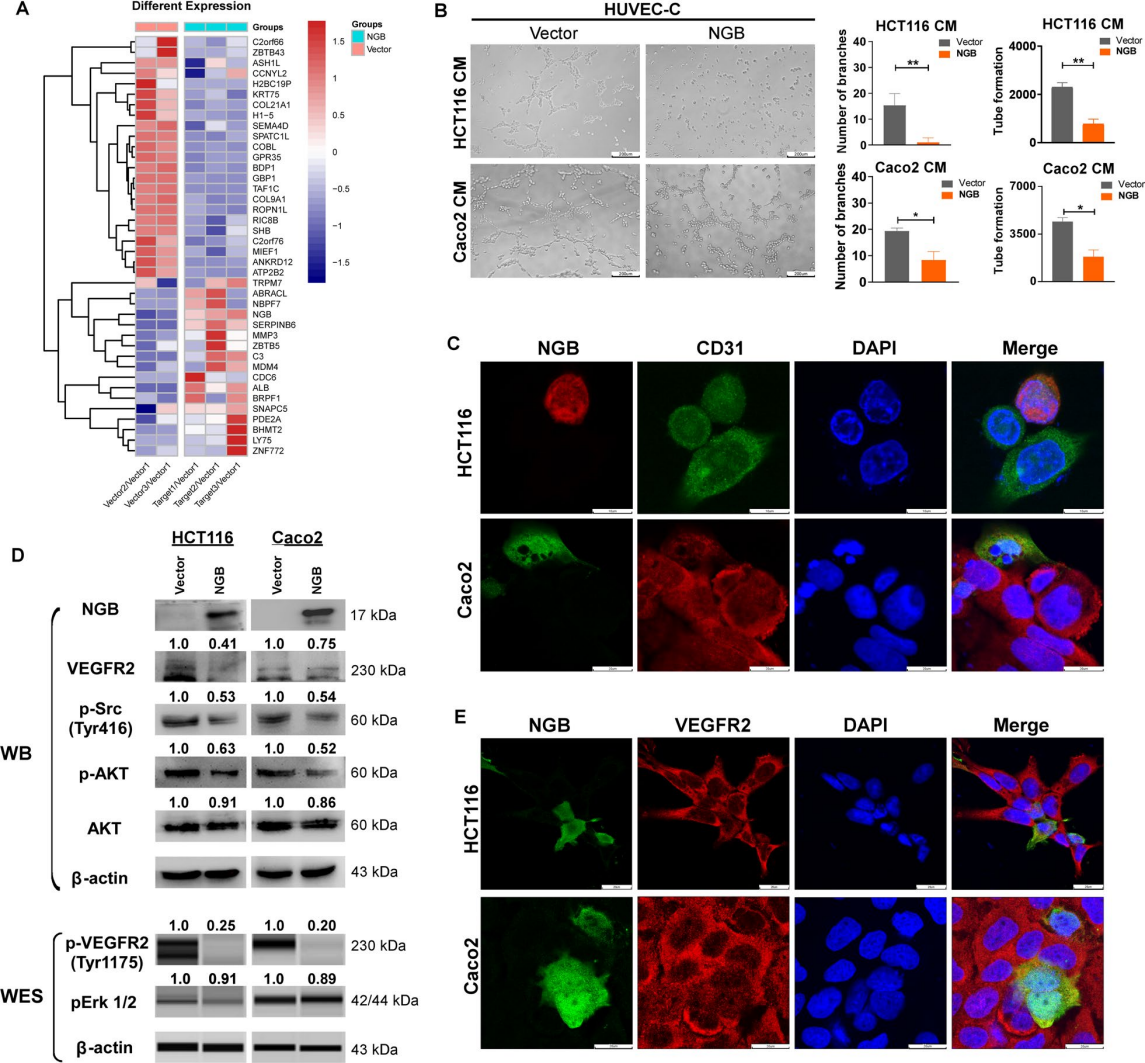
NGB-OE suppresses tumor angiogenesis ability of CRC cells. **A** Heatmap of differentially expressed proteins in NGB-overexpressing or vector HCT116 cells. **B** Angiogenesis estimated by endothelial cell tube formation assay; statistical analysis of tube formation and branches is shown on the right used angiogenesis tool of image J, *N* =  3. CM means conditional medium. **C** Immunofluorescence: the effect of NGB on CD31 expression status in CRC cells. **D** The status of angiogenesis pathway detected by WB or WES. **E** Immunofluorescence: the effect of NGB on VEGFR2 expression in CRC cells. All experiments were performed in triplicate. *p < 0.05, **p < 0.01, ***p < 0.001

In addition, the threshold of DEPs fold change identified 23 downregulated proteins and 17 upregulated proteins (|fold change|> 1.5, Q value < 0.05) (Fig. 4A), of which approximately 37.5% (15/40) were related to cell–cell adhesion, invasion, tumor vessel formation, and the tumor microenvironment (TME). Gene Set Enrichment Analysis (GSEA) revealed that NGB was negatively enriched in epithelial–mesenchymal transition (EMT) pathway (NES = −1.615, *p*-Value = 0.003), regulation of blood vessel endothelial cell migration (NES = −1.552, *p*-Value = 0.014), and VEGFA VEGFR2 (VEGF receptor 2) signaling pathway (NES = −2.36, *p*-Value < 0.001) (Additional file 1: Fig. S5E) compared with vector group. Negative correlation between NGB and regulation pathway revealed by GSEA was consistent with NGB suppress endothelial cell migration observed in xenograft model (Additional file 1: Fig. S4C, D). Consideration of tumor angiogenesis is the nutrition source for tumor invasion and migration in the TME; an endothelial tube formation assay was performed to evaluate angiogenesis. The results showed that the branches and tube formation of HUVEC-C cell were smaller in the NGB-OE medium culture group than in the control group by endothelial cell tube formation experiments (Fig. 4B, *p* < 0.05). Cell viability and migration ability of HUVEC-C were decreased by cultured with NGB-OE cell conditional medium (CM) (Additional file 1: Fig. S6A, B). Immunofluorescence (IF) analysis showed that CD31 was downregulated at 48 h after NGB transfection in HCT116 and Caco2 cells (Fig. 4C). Western blot analysis showed that activation factors of the tumor angiogenesis pathway were downregulated following NGB-OE in HCT116 and Caco2 cells, including VEGFR2, p-VEGFR2 (Tyr1175), p-Src (Tyr416), p-AKT, and pErk1/2, but not AKT (Fig. 4D). IF results implying the regulation of VEGFR2 by NGB were consistent with Western blot tendency (Fig. 4E). These data demonstrated that overexpression of NGB inhibited tumor angiogenesis pathway in HCT116 and Caco2 cells.

### NGB interacts with and downregulates GPR35

**Fig. 5:**
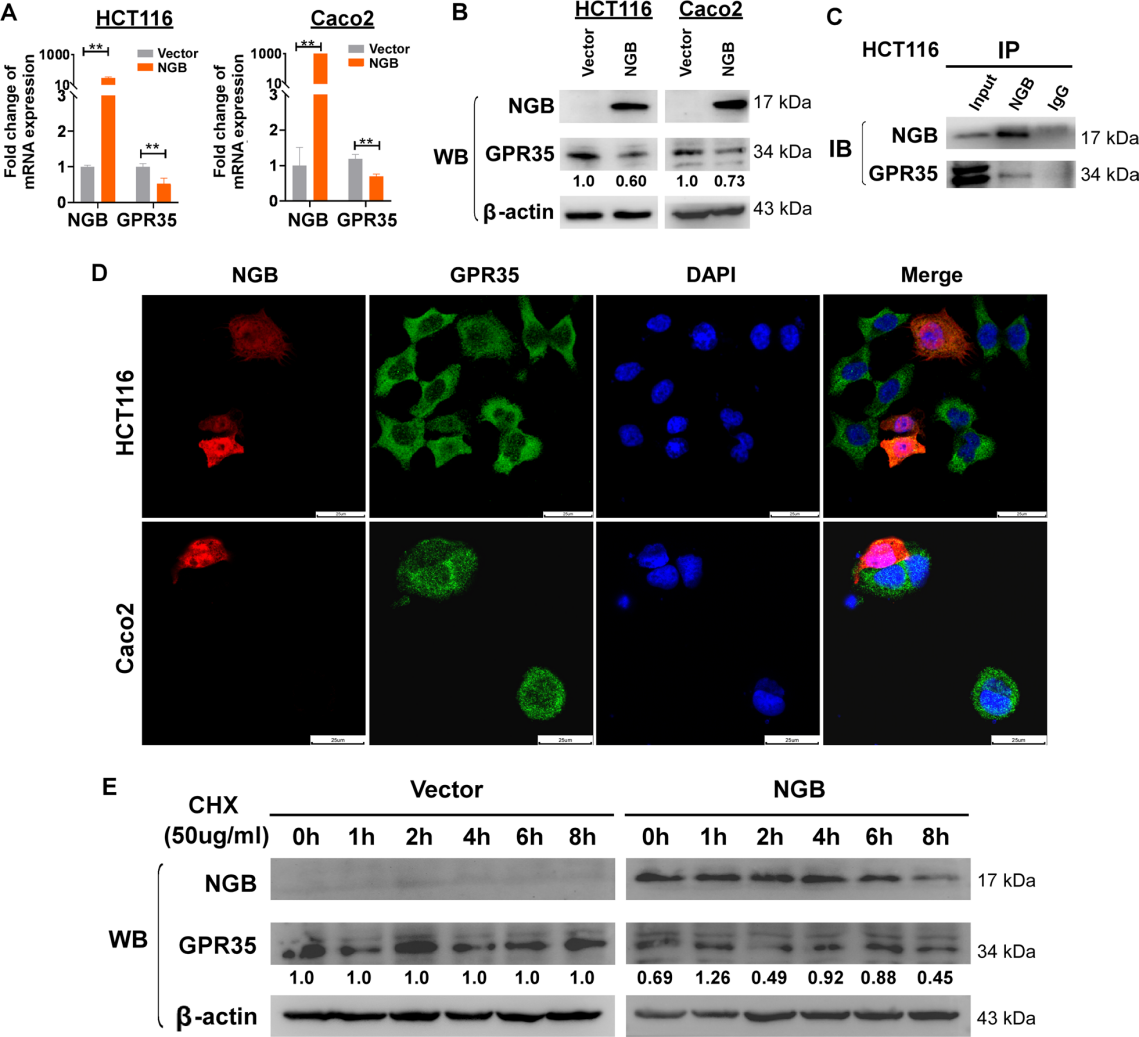
NGB downregulates GPR35 by promoting its degradation. **A**, **B** The expression level of NGB and GPR35 explored by q-PCR and WB. **C** Protein interaction between NGB and GPR35 confirmed by IP. **D** IF: The effect of NGB on GPR35 expression in HCT116 and Caco2 cells. **E** The protein stability of GPR35 after CHX treatment was examined in NGB-OE or vector HCT116 cells. All experiments were performed in triplicate. *p < 0.05, **p < 0.01, ***p < 0.001

Considering the inhibitory effect of NGB on tumor neovascularization in vitro and in vivo (Fig. 4, Additional file 1: Fig. S4C, D, S5E), we decided on investigating its underlying mechanism. Identified from mass spectrometry. GPR35, an important regulator in neovascularization, scored high as NGB negatively correlated protein and thus a perfect candidate for downstream exploration (Fig. 4A, Additional file 1: Fig. S7). Because GPR35 plays an important role in neovascularization [19], we focused on this protein as a downstream target for further study. qRT-PCR and Western blot analysis indicated that NGB overexpression downregulated GPR35 in HCT116 and Caco2 cells on both transcriptional and translational levels (Fig. 5A, B). Immunoprecipitation (IP) assay using anti-NGB antibodies indicated that GPR35 was an interacting protein for NGB (Fig. 5C). IF assay demonstrated that NGB overexpression downregulated GPR35 in HCT116 and Caco2 cells (Fig. 5D). In HCT116 cells treated with cycloheximide (CHX, 50 µg/ml), NGB disrupted the protein stability of GPR35 (Fig. 5E). Taken together, these results suggested that NGB and GPR35 interact mutually, and NGB promotes the degradation of GPR35 protein, thereby much likely to decrease its half-life.

### Ectopic expression of GPR35 reverses the NGB-induced tumor angiogenesis suppression in CRC cells

**Fig. 6:**
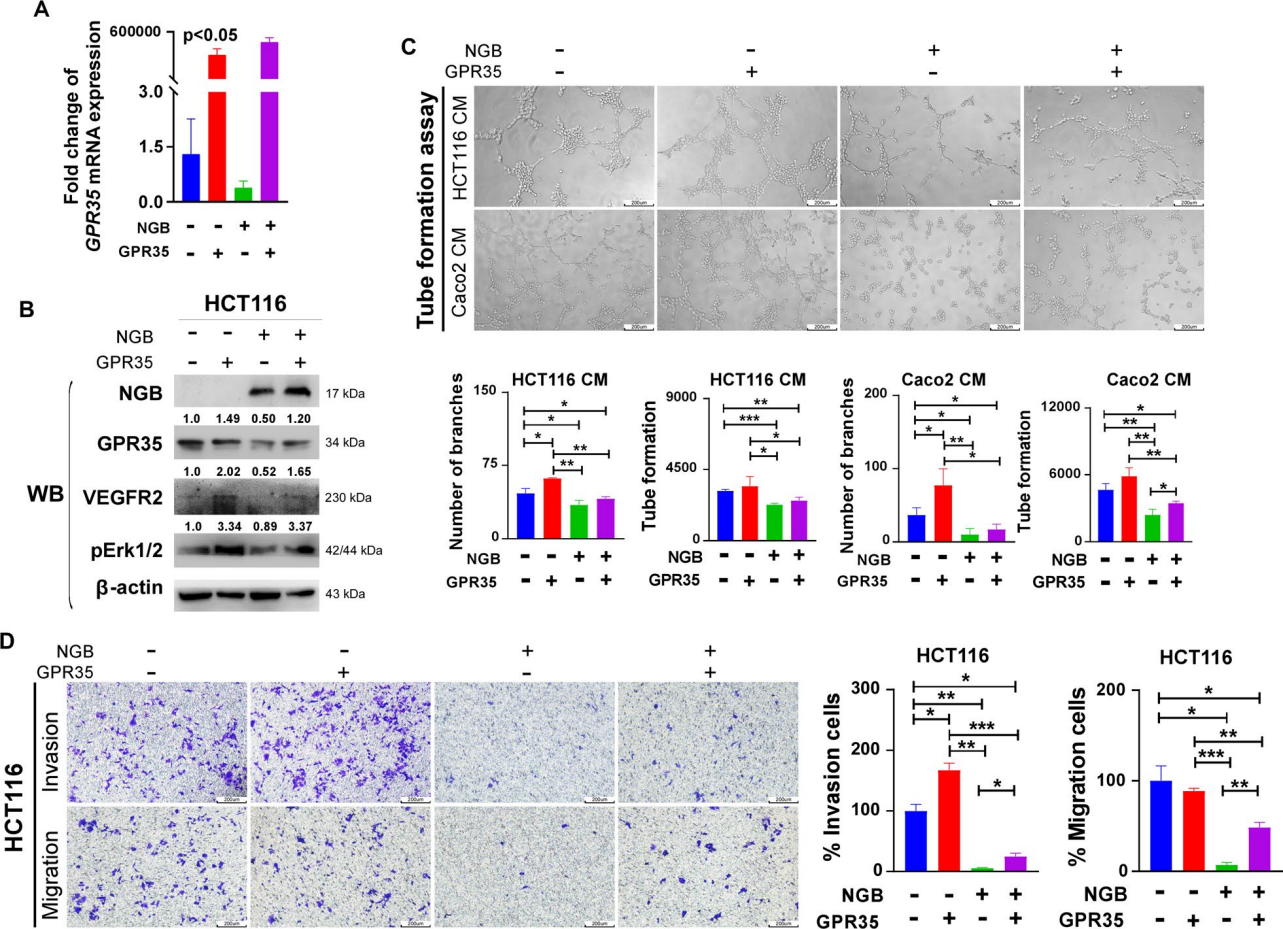
NGB-OE inhibits tumor angiogenesis and cell metastasis, which can be rescued by GPR35 re-expression. **A** The mRNA expression level of GPR35 verified by q-PCR. **B** Changes of activation factors of angiogenesis pathway examined by WB. **C** Tube formation restored by GPR35 in NGB overexpression in CRC cells. Statistical analysis of formation and branch counts is shown below. **D** Cell migration and invasion ability restored by GPR35 overexpression in NGB-OE HCT116 cells. Statistical graphs are shown on the right. All experiments were performed in triplicate. *p < 0.05, **p < 0.01, ***p < 0.001

GPR35 is an upstream factor of angiogenesis in the TME [19]. However, upstream factors controlling over GPR35 remain unreported. We herein provide evidence showing that NGB interacts with and induces the degradation of GPR35 (Fig. 5). To examine the effect of the NGB-mediated downregulation of GPR35 on tumor angiogenesis, we performed rescue experiments in HCT116 cells. GPR35 mRNA and protein expression were rescued in stable NGB-OE HCT116 cells (Fig. 6A, B). As shown in lane 2 of Fig. 6B, the protein levels of VEGFR2 and pErk1/2 were increased by GPR35 restoration. By contrast, the protein levels of VEGFR2 still maintain decreased in the NGB-OE group although GPR35 was rescued in lane 4. Tumor angiogenesis was activated in response to GPR35 restoration in the NGB absent group. However, the branches and tube formation remained suppressed in the NGB-OE medium culture group (Fig. 6C). Moreover, the invasion and migration ability decreased by NGB overexpression were rescued by GPR35 re-expression (Fig. 6D). Taken together, these results demonstrated that NGB suppresses tumor metastasis by downregulating GPR35 /angiogenesis axis probably.

## Discussion

CRC is the fourth most frequently diagnosed cancer and the second leading cause of cancer death [25]. Early diagnose through screening for CRC biomarkers is an effective way to prevent mortality of patients. Nevertheless, due to its heterogeneity, accurate early detection of CRC is essential for therapeutic efficacy improvement [25]. As a frequent event constantly occurring in the early stage of tumors, DNA methylation has the potential application prospects in diagnosis [26].

NGB is the protein-coding gene on chromosome 14q24.3, the third member of the globin family. There had been some literature reports on NGB in neurological diseases and non-neurological tumors [13–17, 27, 28]. In the nervous system, overexpression of NGB protected neurons against mitochondrial dysfunction and neurodegenerative diseases such as Alzheimer's disease as well as acting as a shield in cancer cells [13, 14, 27, 28]. In lung cancer, the upregulation of NGB mRNA was associated with the increase of the hypoxia-inducing factor HIF1, revealing that NGB may be regulated in a hypoxic-dependent manner as a defense mechanism to enable cancer cells to adapt to the tumor microenvironment [17]. However, in breast cancer, hypoxia treatment had no effect on the expression of NGB protein [15]. In addition, it had been reported that HCC cells overexpressed with NGB have a tumor-suppressive effect, in which NGB plays an important role in cell proliferation as the connection between O_2_/ROS signals and intracellular signals [16]. Thus, it can be seen that the biological function of NGB is different in different cancer cells or tissues. But, the function of NGB in colorectal cancer is still unclear, especially the role of epigenetics in regulating NGB.

In this study, we investigated the potential correlation between epigenetically regulation and function of NGB in CRC, in an attempt to explore the complex function of NGB in cancers. Data from TCGA online database combined with our experiments using clinical samples and CRC cell lines showed that NGB was epigenetically silenced in tumor tissues especially lower in liver metastasis tissues, suggested low NGB expression was correlated with metastasis in CRC. Overexpression NGB decreased cell proliferation, and increased cell apoptosis, and caused cell cycle arrest at G2/M phase in HCT116 and Caco2 cells. These results suggested that NGB is a potential marker for patient prognosis appraisal during clinical treatment.

Quantitative proteomics iTARQ analysis was used to explore relevant mechanisms, and the results showed that signal transduction mechanisms and the cytoskeleton were potentially regulated by NGB. GSEA analysis indicated that NGB was negatively enriched in EMT pathway, regulation of blood vessel endothelial cell migration and VEGFA VEGFR2 signaling pathway compared with vector group in CRC cells. Analysis of DEPs showed that approximately 37.5% proteins identified were related to tumor vessel, invasion and the TME. Tumor angiogenesis plays an important role in the growth and metastasis of tumor cells, necessary for the survival and development of tumors. Neovascularization is a characteristic of the TME, where tumor cells promote angiogenesis and inflammation, providing a favorable environment for tumor survival and prosperity. Several pro-angiogenic factors are secreted from tumor cells, tumor-infiltrating lymphocytes or macrophages, which can activate pro-angiogenic signaling pathways, henceforth promoting tumor angiogenesis, growth, and metastasis. For example, VEGFR2 is a core regulator of pro-angiogenic VEGF and angiopoietin signaling. In this study, tube formation CCK8 and Transwell assays confirmed that NGB inhibited cell metastasis by suppressing tumor angiogenesis. Western blot and IF verified that VEGFR2 and tumor angiogenesis activation markers (p-VEGFR, pErk1/2, p-Src, p-AKT) were downregulated in CRC cell lines overexpressing NGB. Tumors harvested from nude mice in the targeted group were significantly smaller because of aberrant NGB expression. Histopathology showed that inflammatory infiltration and endothelial cell infiltration were milder in nude mice from NGB-OE group than the vector group. These results suggested that NGB prevents tumor progression by inhibiting neovascularization in CRC cells.

Online database analysis and iTARQ assays were used to identify downstream targets of NGB related to tumor angiogenesis. GPR35, which scored high in the iTARQ assay, was negatively correlated with NGB and related to tumor angiogenesis. GPR35, a G protein-coupled receptor (GPCR), possesses seven transmembrane domains. Although kynurenic acid and the chemokine CXCL17 [29] were considered candidate ligands of GPR35, a study suggested that GPR35 is an orphan GPCR that interacts with the sodium–potassium pump (Na/K-ATPase) [30]. Activation of the GPR35 signaling pathway promotes tumor angiogenesis in the TME, and loss of GPR35 in macrophages reduces matrix metalloproteinase activity in tumor tissues, a prerequisite for tissue remodeling and angiogenesis. The role of GPR35 as an oncogene in colorectal disease has been defined, although its upstream mechanism remains unclear. In this study, we showed a negative correlation between NGB and GPR35 at the mRNA and protein levels, further confirmed by online database, iTARQ, qRT-PCR, WB, and IF. IF and IP assays demonstrated the presence of protein–protein interaction between NGB and GPR35. The results seem that GPR35 could be involved in the NGB-OE phenotype, NGB-suppressed tumor angiogenesis and cell metastasis by promoting GPR35 degradation. However, since we did not achieve a complete rescue, other mechanisms will surely be involved in determining the phenotype; deeply mechanism needs to explore.

## Conclusion

In conclusion, we showed that *NGB* is epigenetically silenced and acts as a new tumor-suppressor gene in CRC. Distinguished difference of promoter hypermethylation status between tumor and adjacent is identified in this study, implying that NGB could be considered as a predictive biomarker for early diagnoses for CRC. The underlying mechanism includes inhibition of CRC metastasis through suppressing tumor angiogenesis, which is achieved by promoting GPR35 protein degradation in the TME. NGB could be a promising strategy for cancer therapy by decreasing tumor metastasis through the suppression of GPR35/angiogenesis axis. In addition, we noted that NGB inhibits metastasis in CRC not only tumor angiogenesis but also invasion/migration pathway, and we will make efforts to explore in future research work.

## Methods

### Cell culture and tumor samples

The CRC cells used in this study including HCT116, HT-29, LoVo, SW480, Caco2, HCT-15, RKO and HUVECs were purchased from ATCC. Cells were maintained in RPMI 1640 (Gibco-BRL, Germany) supplemented with 10% fetal bovine serum (FBS, Biological Industries BI, Israel). Cells were cultured in a moist environment containing 5% CO_2_ at 37 °C. CRC tissues and paired surgical margin tissues were obtained after surgical procedures conducted at Chongqing University Cancer Hospital, Chongqing, China.

### RNA extraction, RT-PCR and quantitative RT-PCR (qRT-PCR)

Total RNA from cells or tissues was isolated using the TRIzol reagent (Invitrogen, USA) according to the manufacturer’s protocol. Aliquots containing 1 μg of total RNA were reverse-transcribed to 20 μl cDNA using Promega GoScript™ reverse transcriptase (Promega, USA). Reverse transcription polymerase chain reaction (RT-PCR) was performed with Go-Taq (Promega, USA) and the GeneAmp RNA PCR system (Applied Biosystems), and qRT-PCR was performed with ABI 7500 Real-Time PCR System using GoTaq® qPCR Master Mix as reported in our previous study [31]. The primer pairs are listed in Additional file 1: Table S1. All experiments were performed in triplicate.

### Methylation-specific PCR (MSP) assay

Methylation status of NGB was evaluated by MSP assay as previously described [9]. MSP was conducted for 40 amplification cycles using AmpliTaq®-Gold DNA polymerase (Applied Biosystems), with annealing temperatures at 60 °C and 58 °C for methylated and unmethylated samples, respectively. Electrophoreses on 2% agarose gels of PCR products were then performed. Gel imaging system (Bio-RAD Gel Doc XR + , USA) was used to visualize all the MSP results.

### DNA methylation analysis

DNA methylation status of NGB promoter in CRC tissues was analyzed by Methyltarget (Genesky Biotechnology Inc., Shanghai, China) sequencing on Illumina MiSeq platform according to the manufacturer's protocols. The beta value indicates the level of DNA methylation ranging from 0 to 1 (full methylated); different beta value cutoffs have been considered to indicate unmethylation [Beta value: 0–0.2], hypo-methylation [Beta value: 0.3–0.25], hyper-methylation [Beta value: 0.7–0.5]. The coverage and quality statistics of the targeted bisulfite sequencing analysis are for each sample summarized in Additional file 2. And the frequency of NGB methylated in CRC tissues detected by MSP. All experiments were performed in triplicate. The primers used for MSP are listed in Additional file 1: Table S1.

### Plasmid and stable cell line construction

The pCMV6-Entry-NGB (OriGene Technologies), pReceiver-M02-GPR35 (Genecopoeia) and pCMV6-Entry plasmids were transfected into HCT116 and Caco2 cells using Lipofectamine 2000 (Invitrogen, USA) according to the manufacturer’s protocol. Cells were grown in non-selective growth medium for 48 h after transfection, and the medium was then replaced with selection medium containing 8 μl/ml G-418 (50 mg/ml) for HCT116 or 7 μl/ml for Caco2 and cultured for another 14 days. Overexpression of NGB was confirmed by RT-PCR and Western blot before other experimental procedures [31]. All experiments were performed in triplicate.

### 5-Aza-2’-deoxycytidine (Aza) treatment assay

Incorporation of Aza into DNA of cultured cells leads to rapid loss of DNA (cytosine-C5) methyltransferase (DNA (C5) MTase, Dnmt) activity and typically used to active gene expression by promotor demethylation. Stable expressed NGB/Vector HCT116 and Caco2 cells were treated with 10 μM Aza for 72 h; then, cells were harvested for qPCR and MSP. All experiments were performed in triplicate.

### Cell counting kit 8 (CCK8) assay

Cell proliferation was evaluated by the CellTiter 96 AQueous One Solution Cell Proliferation Assay (CCK8, Promega) according to the manufacturer’s instructions. Cells were seeded on 96-well plates (2000 cells per well) with 200 μl medium containing 10% FBS, cultured for 0, 24, 48, 72 h, and then incubated with 100 μl medium containing 10 μl CellTiter 96 Aqueous One Solution reagent for 2 h at 37 °C in the dark. Absorbance was measured at 450 nm with a microplate reader (Multiskan MK3, Germany). Each experiment was repeated three times.

### Colony formation assay

HCT116 and Caco2 cells stably expressing *NGB* or vector were seeded in 6-well plates at ascendant densities of cells per well. After 14 days of culture, surviving colonies (≥ 50 cells/colony) were counted after fixation and staining with 1% crystal violet. All experiments were performed in triplicate.

### Flow cytometry analysis for cell cycle and apoptosis assays

To analyze cell cycle status, cells were harvested and fixed in ice-cold 70% ethanol overnight ahead and then washed with PBS twice, treated with 5 mg/ml RNase A (Sigma) at 37 °C in the dark for 30 min, and stained with propidium iodide (PI) for 30 min at room temperature. For apoptosis assays, cells were stained with annexin V-fluorescein isothiocyanate (FITC) and PI. Data on the cell cycle status and apoptosis were analyzed using CELL Quest software (BD Biosciences, USA). All experiments were performed in triplicate.

### Transwell assay

Transwell chambers (8 μm pore size, Corning, USA) were used to evaluate cell migration and cell invasion abilities. For invasion assay, chambers were incubated with Corning Matrigel Basement Membrane Matrix (Corning Cat. No. 356234) at 37 °C for 4 h beforehand. Cells stably expressing *NGB* or vector were harvested and re-suspended in serum-free medium. The cell suspension was added to the upper chamber (3 × 10^4^ cells per chamber for HCT116, 4 × 10^4^ cells per chamber for Caco2), and the lower chamber was filled with 600 μl medium containing 20% FBS. After 48-h cultivation, cells were fixed with 4% paraformaldehyde and stained with 1% crystal violet. Migrated cells were photographed under a microscope magnification. All assays were performed three times.

### iTRAQ quantitative proteomics

The NGB expression and vector-transfected cells were harvested for quantitative proteomics analysis by iTRAQ. The procedure involved protein extraction, enzymolysis, iTRAQ labeling, sample mixing, and LC–MS/MS (liquid chromatograph mass spectrometer) analysis. All iTRAQ experimental procedures were finished by BGI Genomics (Shenzhen, China). The mass spectrometry proteomics data have been deposited to the ProteomeXchange Consortium via the PRIDE [32] partner repository with the dataset identifier PXD038420.

### Mouse xenograft model

The endogenous anti-tumor activity of NGB was evaluated using a mouse xenograft model. Vector- and *NGB*-expressing HCT116 cells (2 × 10^6^ in 0.2 ml cold PBS) were injected subcutaneously into the left and right sides of nude mice haunch (male, aged 4–6 weeks, *n* = 5 per group). Tumor volumes were monitored (volume = 0.5 × length × width^2^) after engraftment every two days before the tumor volume exceeded 1cm^3^, and tumor weights of were documented after killing and dissection.

### Hematoxylin–Eosin (H&E) staining

The procedure of H&E staining was as follows: tissue slides were deparaffinized by xylene and hydrated using ethanol with descendant concentrations (100%, 95%, 85%, and 75%) for 5 min and water for 3 min. Nucleus staining in hematoxylin was performed for 3 min, followed by washing in running tap water for 5 min, 1% acid ethanol for a few seconds to induce differentiation, and rinsing in running tap water until cells became blue. Cells were then counterstained in 1% eosin for 10 min. Images were captured under a microscope (Olympus, Japan) after dehydration, clearing, and mounting. All experiments were performed in triplicate.

### Immunohistochemistry

Immunohistochemistry was performed using a mouse streptavidin-peroxidase assay system according to our previously described protocol. Three paired human colorectal carcinoma tissues were used to evaluated protein expression level of NGB (sc-133086, 1:100 dilution). Sections of xenograft of nude mice were incubated with primary antibodies against NGB (sc-133086, 1:100 dilution), Ki67 (sc-23900,1:200 dilution), CD8α (ab237710, 1:500 dilution) or CD31 (#3528,1:400 dilution) overnight at 4 °C, incubated with secondary antibody, and stained with diaminobenzidine. The staining was assessed by a trained pathologist using Image-Pro Plus (version 6.0). All assays were performed three times independently.

### Endothelial cell tube formation assay

HUVEC were used for tube formation assay. Matrigel matrix (Corning Cat. No. 356234) was diluted using serum-free RIPM 1640 (50 µl/well), the liquid was pipetted into 96-well plates (carefully avoiding air bubbles), and the plates were incubated at 37 °C for 1 h. HUVEC cell was harvested after 24 h of pre-treatment with NGB- or Vector- HCT116 or Caco2 cells culture medium, and re-suspend with different conditional medium, respectively. Counts and added cells onto the 96-well plates containing matrigel and incubated for 4 h (1.5 × 10^4^ HUVEC per well). Tube formation was observed and pictured under a microscope (Olympus, Japan). All assays were performed three times independently.

### Immunofluorescence (IF)

HCT116 cells were seeded on a microcover slip and then transfected with pCMV6-Entry-NGB plasmids. After 48 h, cells were fixed with 4% paraformaldehyde, permeabilized with 0.5% Triton X-100 and then blocked with blocking buffer. Afterward, slides were incubated with primary antibody at 4 °C. After 20 h, the cells were incubated with Alexa Fluor 594- or 488-conjugated goat anti-mouse and goat anti-rabbit secondary antibodies (ab150113, ab150080, Abcam) for 1 h at room temperature in the dark. All slides were next counterstained with 4'-6-diamidino-2-phenylindole (DAPI, Beyotime Biotechnology). Photomicrographs were captured with a confocal laser scanning microscope. All assays were performed three times. Primary antibodies included anti-NGB (sc-133086, Santa Cruz Biotechnology), anti-GPR35 (TA313953, Origene technologies), anti-Myc tag (#2278; Cell Signaling Technology), anti-CD31 (#3528, Cell Signaling Technology), and anti-VEGFR2 (sc-6251, Santa Cruz Biotechnology).

### Co-Immunoprecipitation (Co-IP) assay

The procedure used for the Co-IP assay was described previously [31]. Beads of MAg25K/Protein A/G (Cat No. LM20210817, Enriching Biotechnology) were used in this experiment.

### Western blot and capillary electrophoresis

BCA protein assay was used to measure protein concentration. Western blot procedure was conducted as previously [31]. Equivalent protein amount (40 μg) was loaded and separated by 8–12% SDS–polyacrylamide gels and then transferred onto Immobilon-P Membranes (PVDF, IPVH00010, ISEQ00010, Millipore). The membranes were incubated at 4 °C overnight with the indicated primary antibodies. After washing three times with PBS containing 10% Tween-20 (PBST), the membranes were incubated with horseradish peroxidase-conjugated goat anti-rabbit or anti-mouse secondary antibodies. SuperSignal™ West Pico PLUS (#34577, Thermo Scientific) was used for visualization. Capillary electrophoresis experiment was performed on WES-Automated Western Blots with Simple Western (Proteinsimple, Bio-techne) following guidelines of sampler kits (12–230 kDa separation module, #SM-W001,). Antibodies against NGB, VEGFR2 (sc-6251), AKT (sc-81434) and β-actin (sc-8432) were purchased from Santa Cruz Biotechnology. Antibodies against p-VEGFR2 (#2478), pAKT (#4060), p-Src (#6943) and p-ERK 1/2 (#4370) were purchased from CST. GPR35 (DF4973, Affinity), E-cadherin (abcam, ab76055), Vimentin (abcam, ab8069), N-cadherin (BD, 2248858). All assays were performed three times independently. 

### Statistical analysis

Image data were quantified by Image J, the original blots are performed in Additional file 3. Data were analyzed using SPSS 22.0 software (SPSS, Inc., Chicago, IL, USA). Continuous variables are reported as the mean ± SD determined by GraphPad Prism 8 (GraphPad Software, Inc., La Jolla, California). Data were analyzed using the Kolmogorov–Smirnov test or Shapiro–Wilk test. Data with a normal distribution were compared with a two-tailed Student’s *t* test, and the Mann–Whitney U test was used for data without normal distribution. Categorical values were compared with the Chi-square or Fisher’s exact test. α = 0.05 was considered as the inspection level.

## Supplementary Information

**Additional file 1.** Supplementary figures and tabel.

**Additional file 2.** Methyl target information and results.

**Additional file 3.** Original blots.

## Data Availability

The raw mass spectrometry data generated in this study have been deposited in the PRIDE database with identifier PXD038420.

## Abbreviations

Aza: 5-Aza-2’-deoxycytidine
CRC: Colorectal cancer
CRC-A: CRC-adjacent
COAD: Colon adenocarcinoma
CCK8: Cell counting kit 8
CYGB: Cytoglobin
CHX: Cycloheximide
Co-IP: Co-Immunoprecipitation
CM: Conditional medium
DEPs: Differentially expressed proteins
EMT: Epithelial–mesenchymal transition
FITC: Fluorescein isothiocyanate
GSEA: Gene set enrichment analysis
GO: Gene ontology
GPCR: G protein-coupled receptor
IHC: Immunohistochemistry
HE: Hematoxylin–eosin
IF: Immunofluorescence
IP: Immunoprecipitation
KOG: Eukaryotic orthologous group
LC–MS: Liquid chromatograph-MS
MS: Mass spectrometer
MSP: Methylation special-PCR
MVD: Microvessel density
NGB: Neuroglobin
NGB-OE: NGB overexpression
OS: Overall survival
PI: Propidium iodide
qRT-PCR: Quantitative RT-PCR
RT-PCR: Reverse transcription polymerase chain reaction
TCGA: The cancer genome atlas
TME: Tumor microenvironment
TPM: Transcripts per million
UALCAN: University of ALabama at Birmingham CANcer data analysis
VEGF: Vascular endothelial growth factor
VEGFR2: VEGF receptor 2
WB: Western blot
WES: Automated Western Blots
KEGG: Kyoto encyclopedia of genes and genomes
