# Supplementary material for: Neuroglobin plays as tumor suppressor by disrupting the stability of GPR35 in colorectal cancer
Source: Clin Epigenetics. 2023 Apr 1;15:57. doi: 10.1186/s13148-023-01472-2 (PMC10067258; doi:10.1186/s13148-023-01472-2)
Supplement: Supplementary file 1 — Additional file 1. Supplementary figures and tabel. [file 13148_2023_1472_MOESM1_ESM.docx]

**Additional file 1**

**Supplementary figures**

**sFigure 1. Online database bioinformation analysis of NGB.** (A) Log 2(TPM+1) expression of NGB in CRC. TPM: Transcripts Per Million, data from GEPIA, http://gepia.cancer-pku.cn/ (B) Effect of NGB expression level on CRC patient survival, data from Kaplan-Meier http://kmplot.com/.

**sFigure 2. Protein expression level of NGB in human normal colon tissues and colorectal adenocarcinoma.** IHC image credit: Human Protein Atlas (HPA), image available from version 22.0.proteinatlas.org.

^1^ https://www.proteinatlas.org/ENSG00000165553-NGB/tissue/colon#imid_17164053. ^2^ https://www.proteinatlas.org/ENSG00000165553-NGB/pathology/colorectal+cancer#imid_17163719.

**sFigure 3. Bio-function of NGB in CRC cells.** (A) Photograph of a colony formation assay. (B-C) Cell cycle and cell apoptosis analyzed by flow cytometry. (D) Metastasis markers evaluated by Wester blotting.

**sFigure 4. Xenograft tumors of NGB-OE/HCT116 in nude mice.**

(A) Histopathology of nude tumor tissues by HE staining. (B) Mean gray value of H&E staining in xenograft tumors. (C) The protein expression of NGB, Ki67, CD31 and CD8α detected by IHC. (D) Microvessel density of xenograft tumors.

**sFigure 5. Enrichment analysis of differentially expressed proteins (DEPs) detected by iTARQ.** (A) Volcano plot of DEPs, FC > 1.2, p < 0.05. FC: fold change. (B) GO enrichment of DEPs. (C) KEGG enrichment analysis of DEPs. (D) KOG enrichment analysis of DEPs. (E) The different functional biological enrichment analyzes by GSEA between *NGB* group and vector group.

**sFigure 6** **Cell viability of endothelial vessel cell were detected by transwell.** (A) Cell viability of HUVEC-C after treatment with NBG-OE or vector CRC cells culture conditional medium (CM). (B) Cell migration ability of HUVEC-C decreased after treatment with NBG-OE or vector CRC cells culture CM. Statistical graphs are shown on the right.

**sFigure 7. The correlation relationship between NGB and GPR35.** (Left) In primary tumor of CRC. (Right) In liver metastasis of CRC. Data from GSE41258.

**sfigure1.**

**
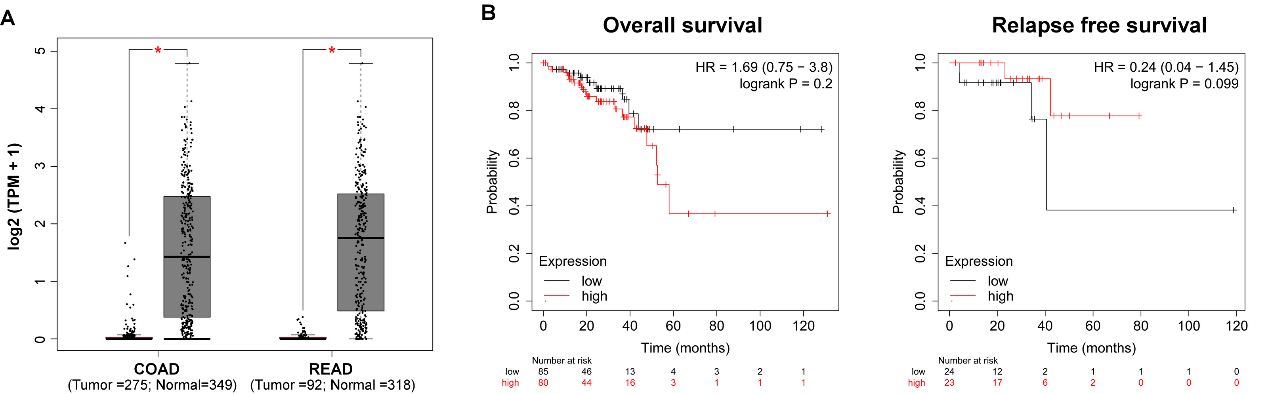
**

**sfigure2.**

**
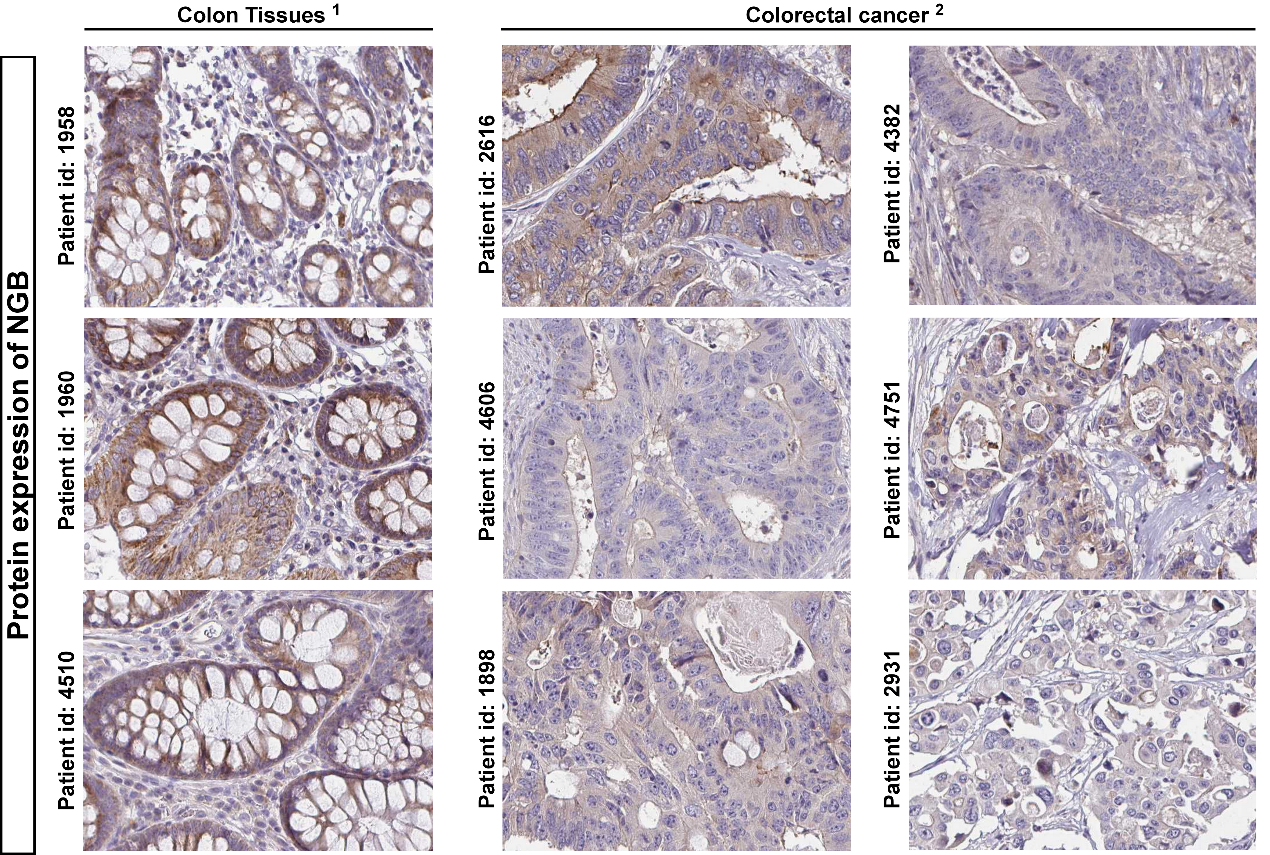
**

**sfigure3.**

**
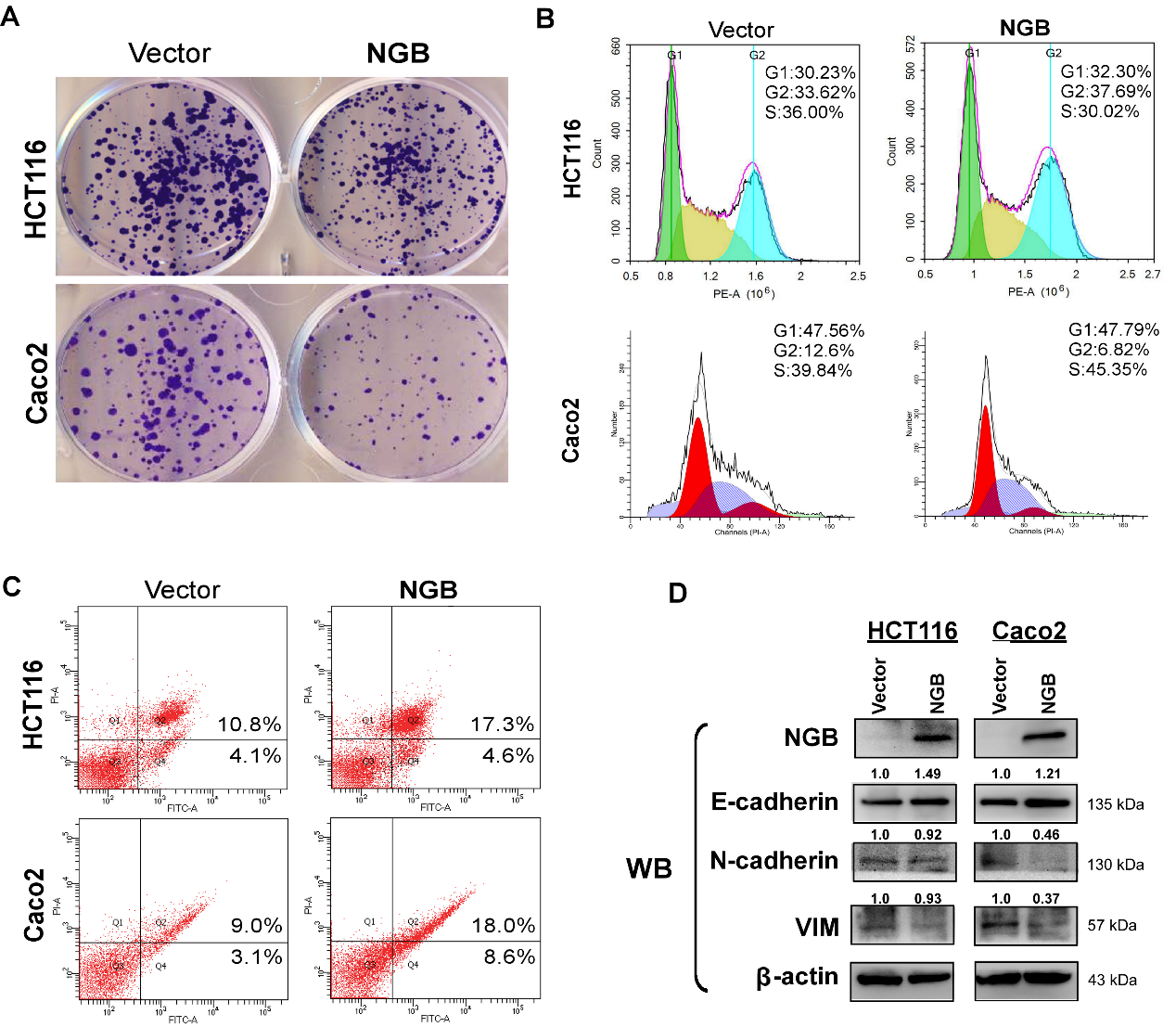
**

**sfigure4.**

**
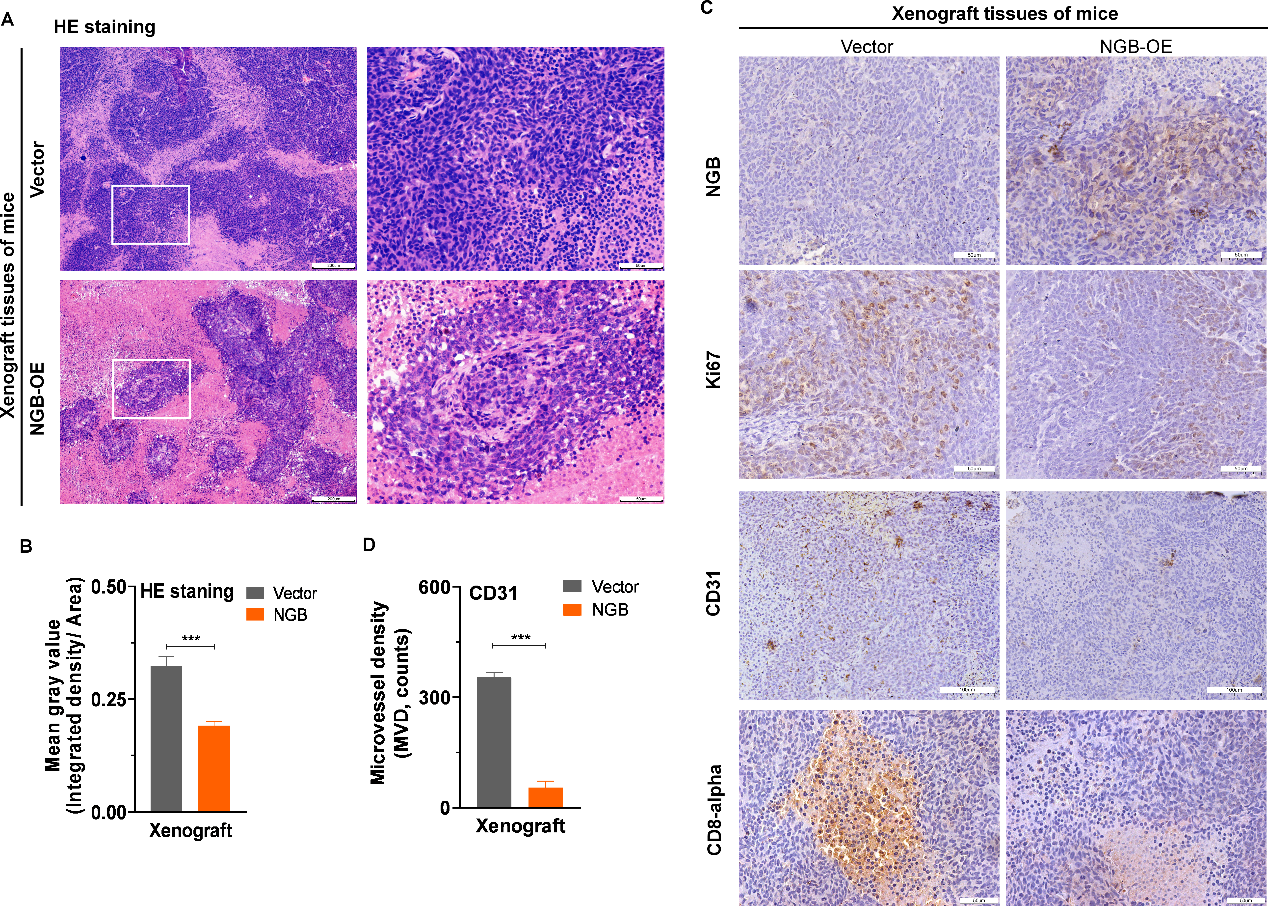
**

**sfigure5.**

**
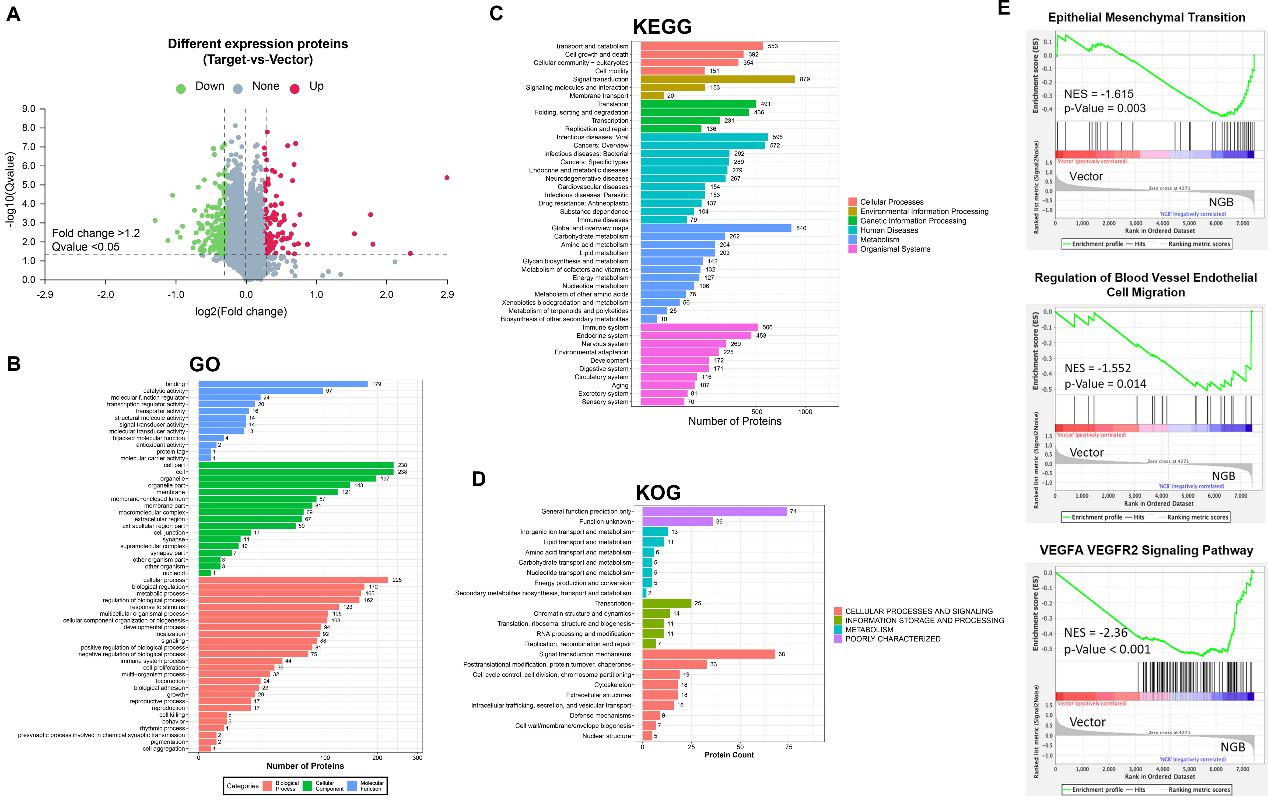
**

**sfigure 6**

**
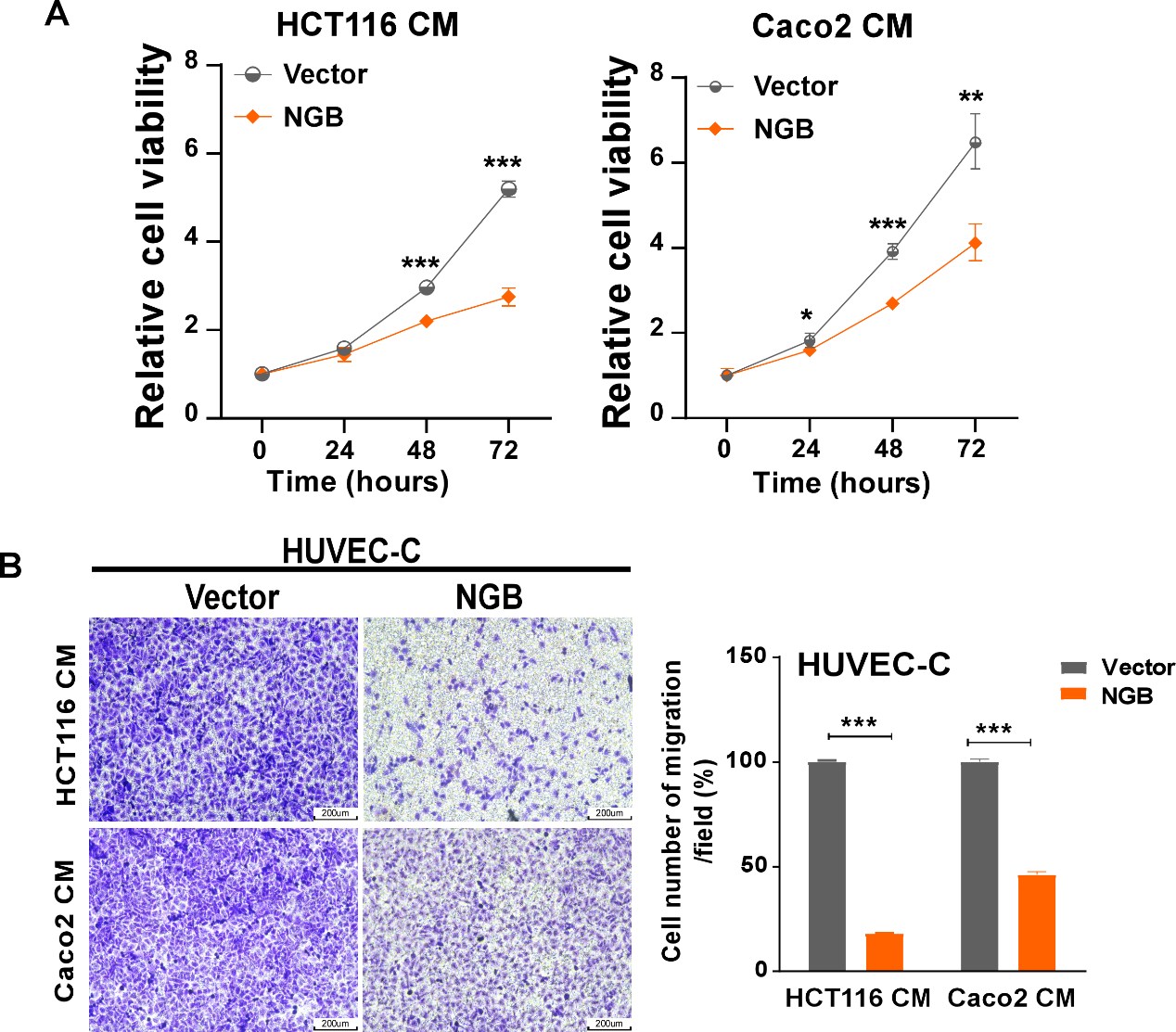
**

**sfigure7**

**
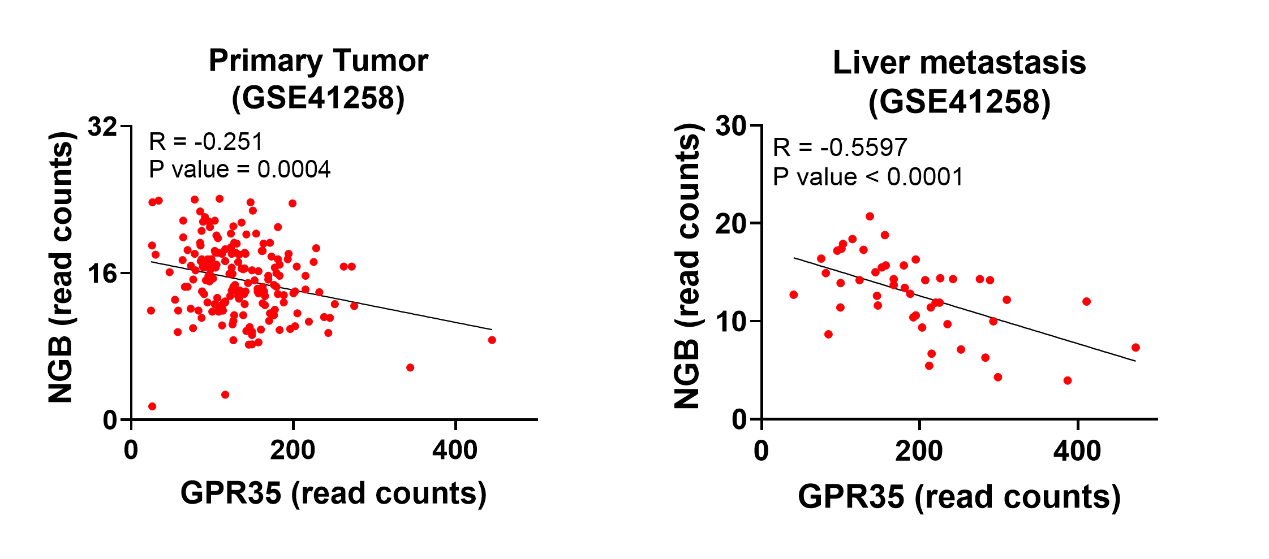
**

**Supplementary Table 1. List of primers used in this study**

| **PCR** | **Primer** | **Sequence (5’-3’)** | **Size (bp)** | **Amplification (cycles)** | **Annealing temperature (℃)** |
| --- | --- | --- | --- | --- | --- |
| RT-PCR | *NGB-F* | TCCAGTACAACTGCCGCCAG | 102bp | 32 | 55 |
|  | *NGB-R* | CTGCAGCATCAATCACGAGC |  |  |  |
|  | *GPR35-F* | GCATCTACATGACCAACCTG | 149bp | 32 | 55 |
|  | *GPR35-R* | GCTGATGCTCATGTACCTGTT |  |  |  |
|  | *GAPDH-F* | GGAGTCAACGGATTTGGT | 206bp | 23 | 55 |
|  | *GAPDH-R* | GTGATGGGATTTCCATTGAT |  |  |  |
| MSP | *NGB-M1* | GTAGGGGTGTATTTCGTTGTC | 106bp | 40 | 60 |
|  | *NGB-M2* | ACCAAACCGCTCACACGCG |  |  |  |
|  | *NGB-U1* | TGTAGGGGTGTATTTTGTTGTT | 109bp | 40 | 58 |
|  | *NGB-U2* | AAACCAAACCACTCACACACA |  |  |  |
| PCR | *β-actin-F* | TCCTGTGGCATCCACGAAACT | 315bp | 23 | 55 |
|  | *β-actin-R* | GAAGCATTTGCGGTGGACGAT |  |  |  |
