# Supplementary material for: Neuroglobin plays as tumor suppressor by disrupting the stability of GPR35 in colorectal cancer
Source: Clin Epigenetics. 2023 Apr 1;15:57. doi: 10.1186/s13148-023-01472-2 (PMC10067258; doi:10.1186/s13148-023-01472-2)

NGB-CRC gel/blots

Figure 1D. RT-PCR

GeneRuler 100 bp Plus DNA
CatNo.SM0322

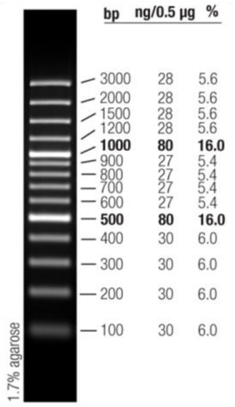

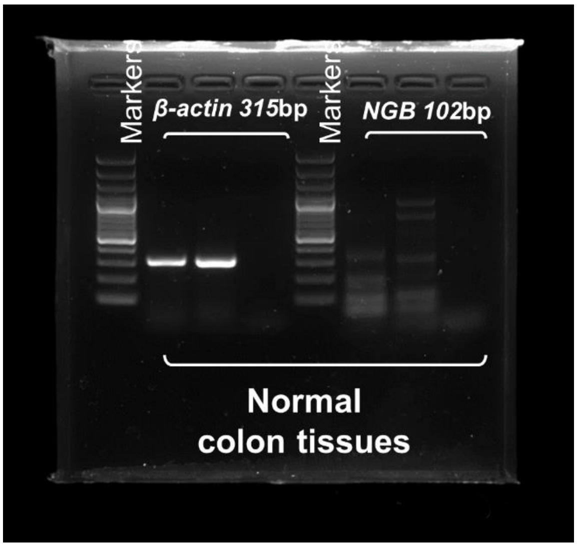

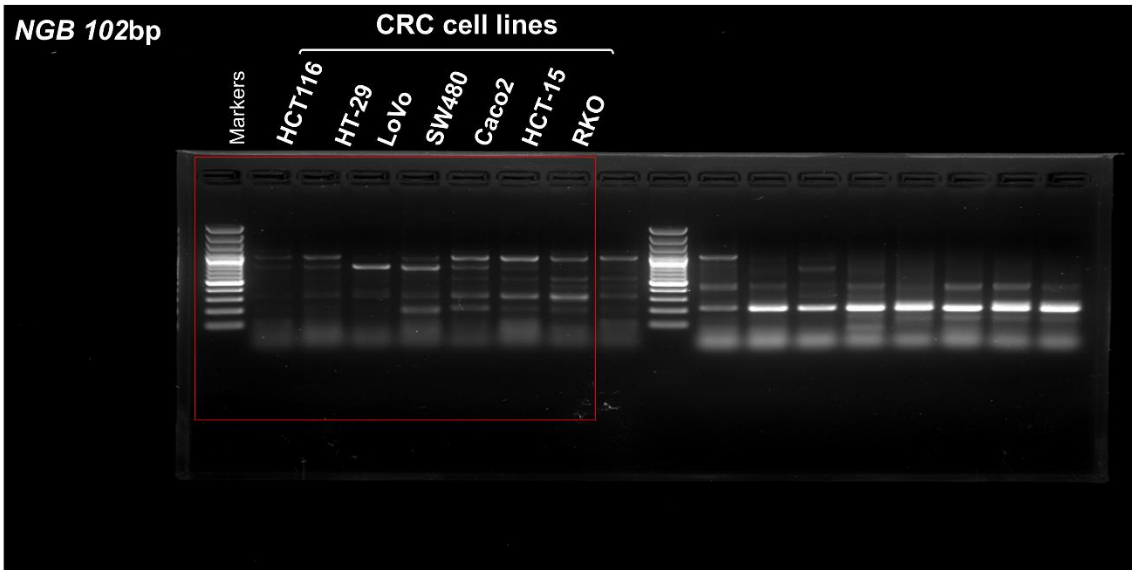

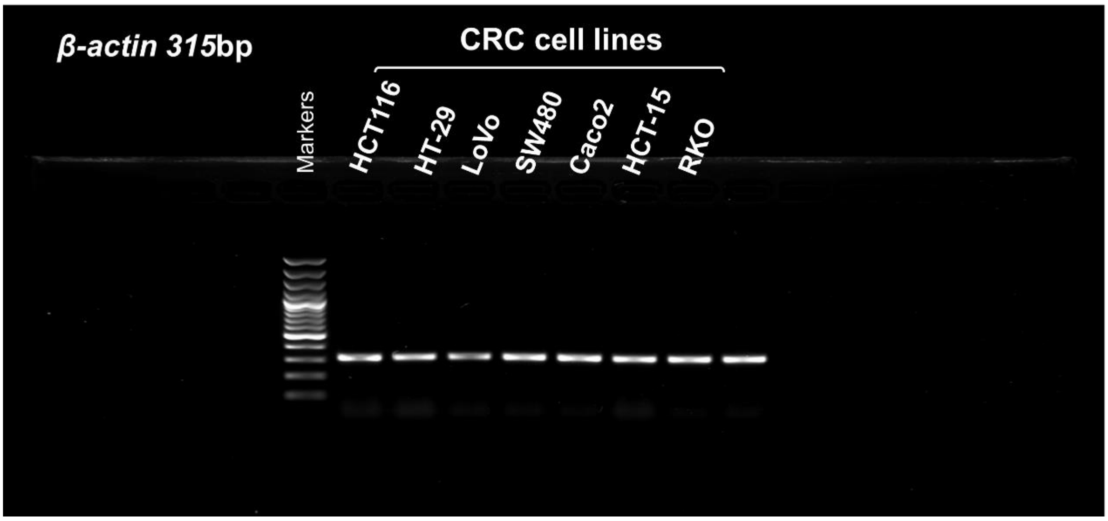

Figure 1D. WB

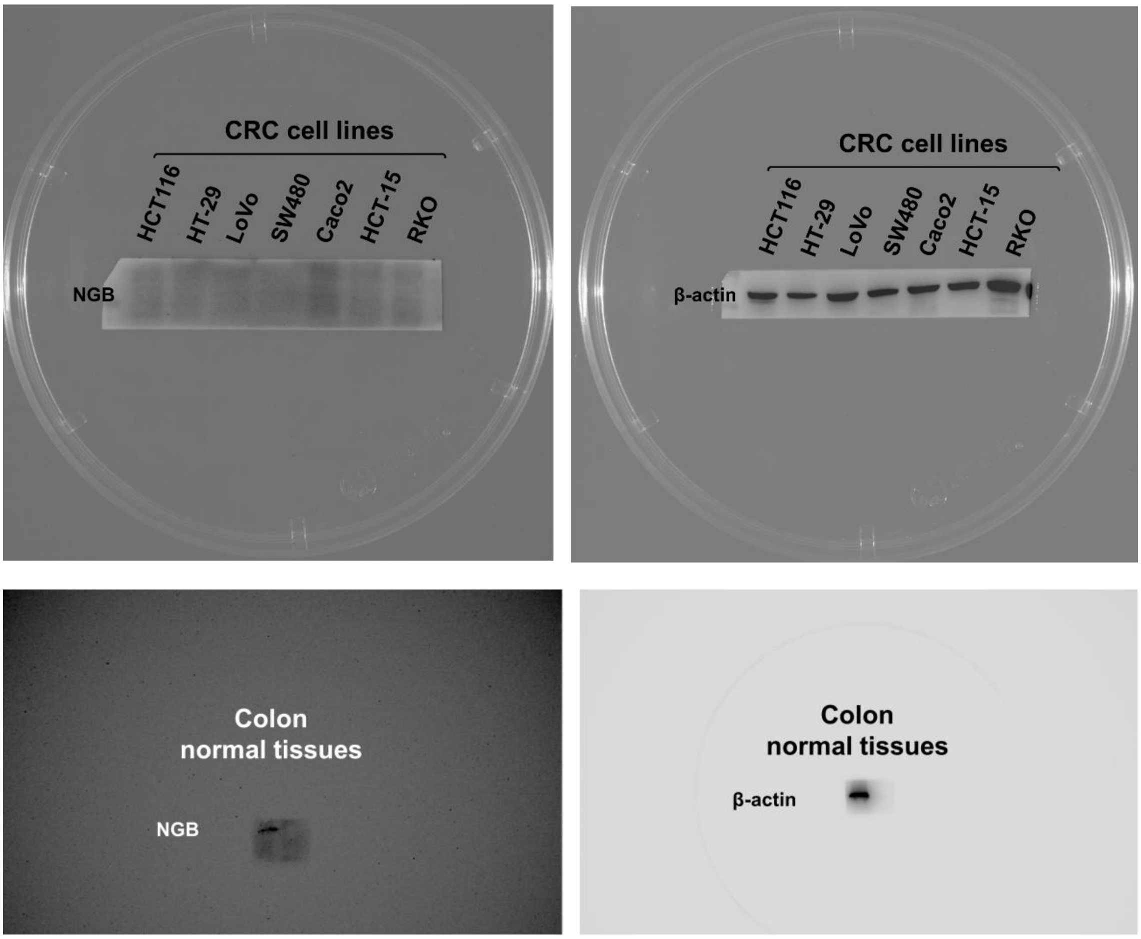
CRC cell lines
HCT116
HT-29
LoVo
SW480
Caco2
HCT-15
RKO
NGB
CRC cell lines
HCT116
HT-29
LoVo
SW480
Caco2
HCT-15
RKO
β-actin
Colon
normal tissues
NGB
Colon
normal tissues
β-actin

Figure 2B. MSP

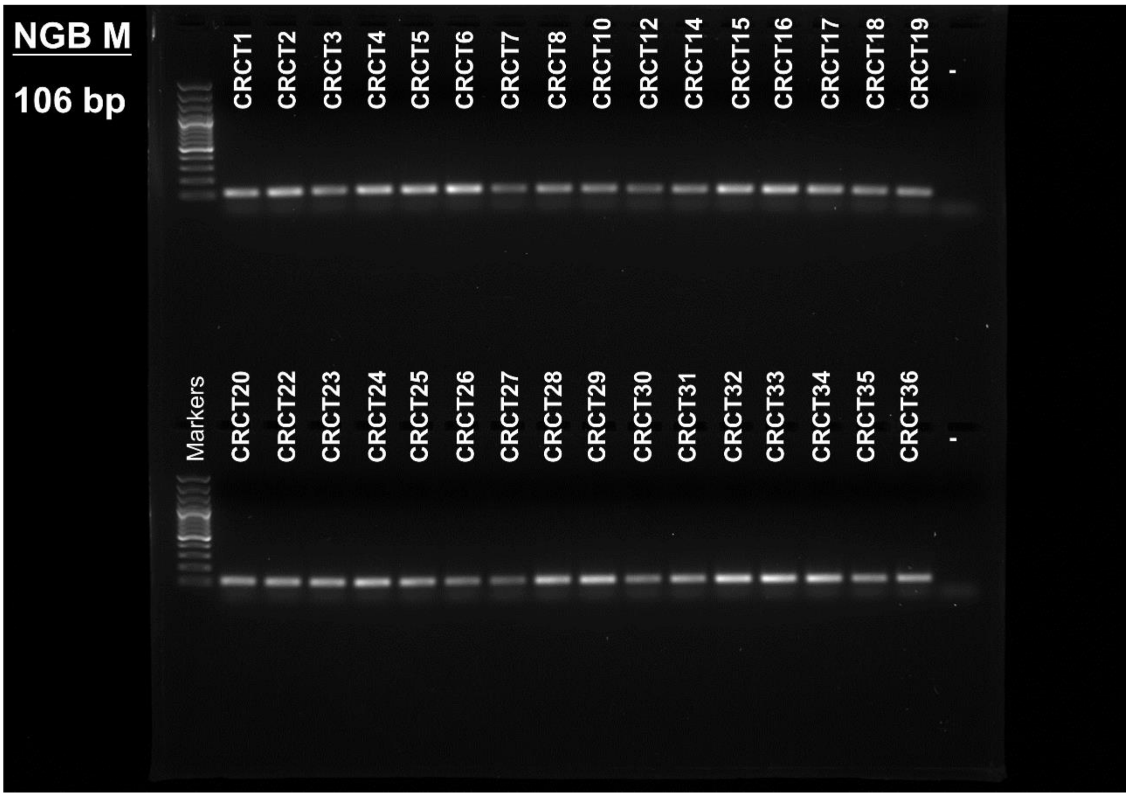

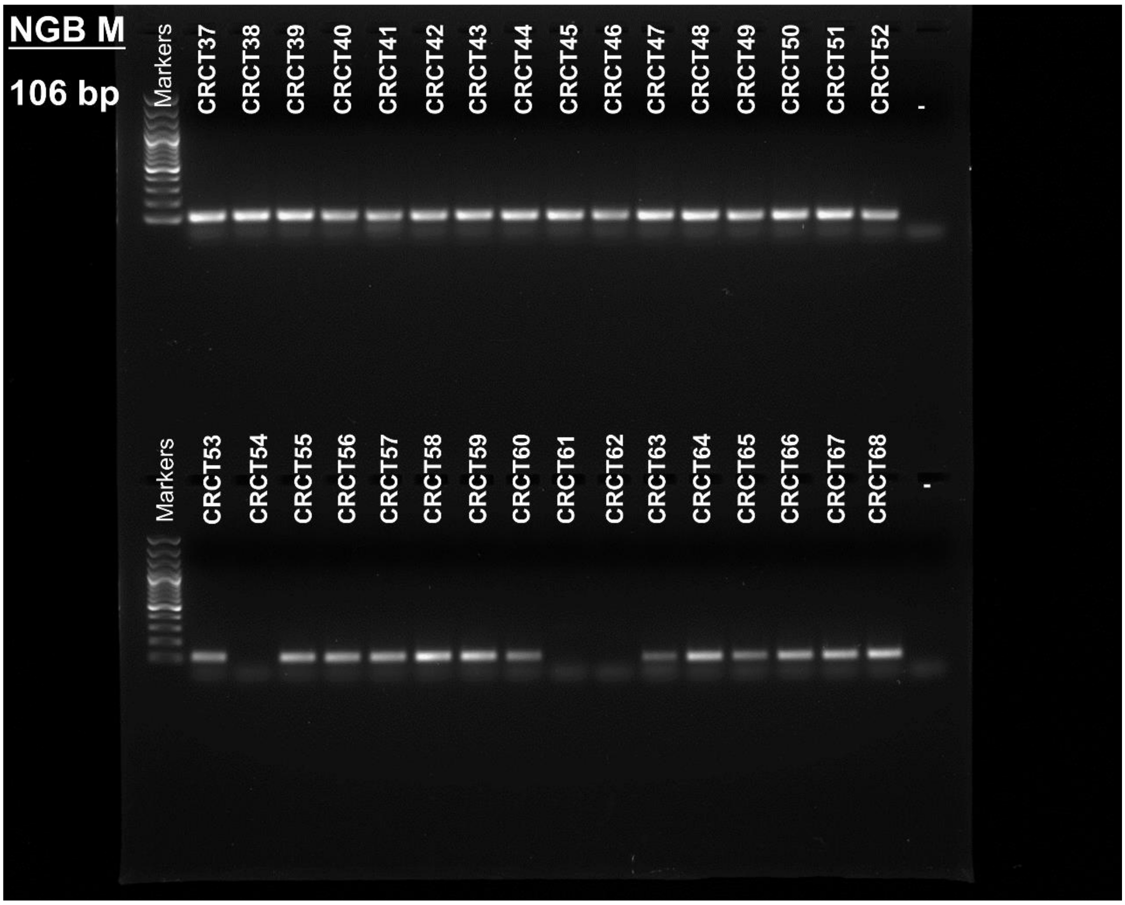

Figure 2B. MSP

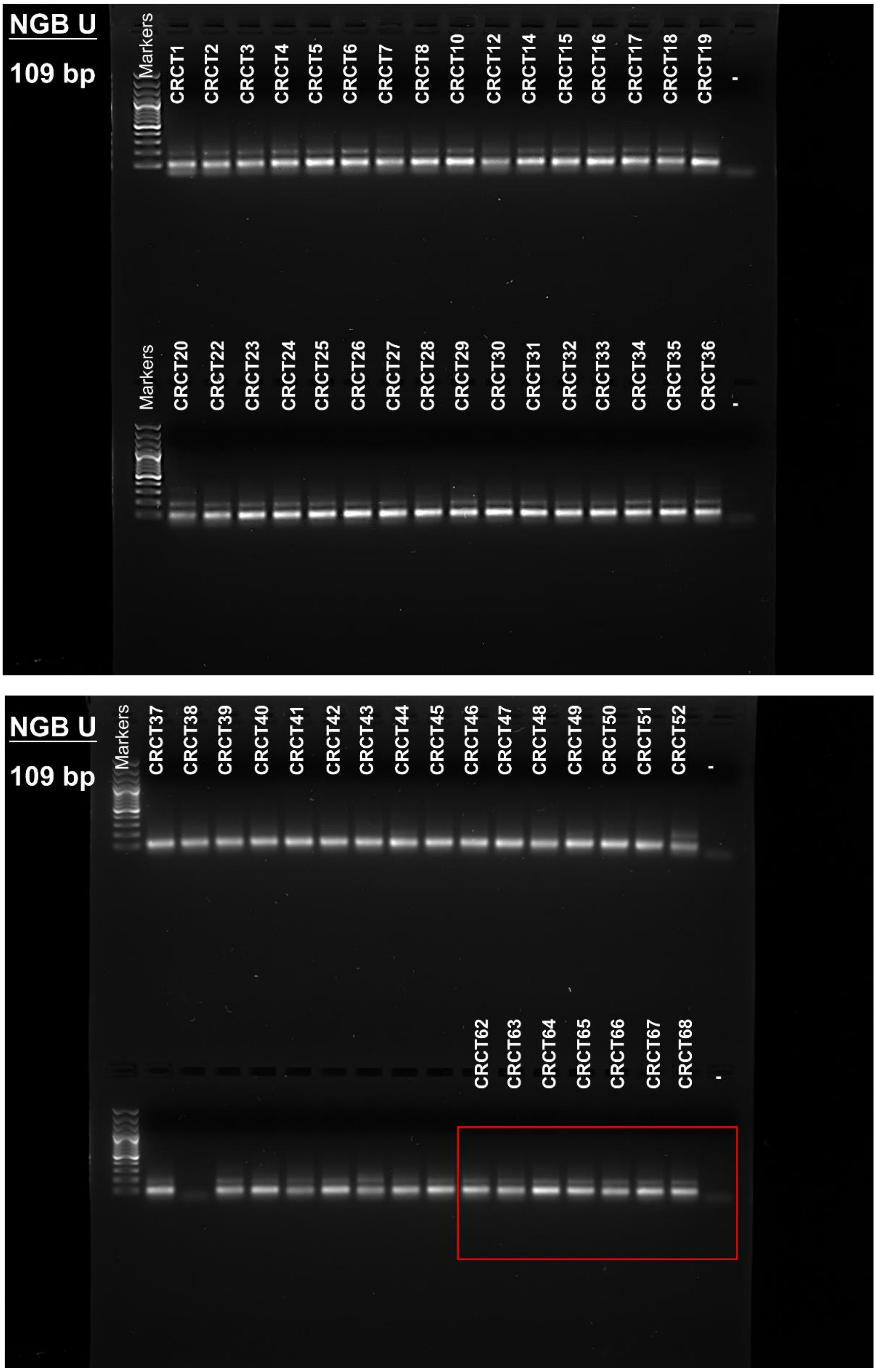

Figure 2B. MSP

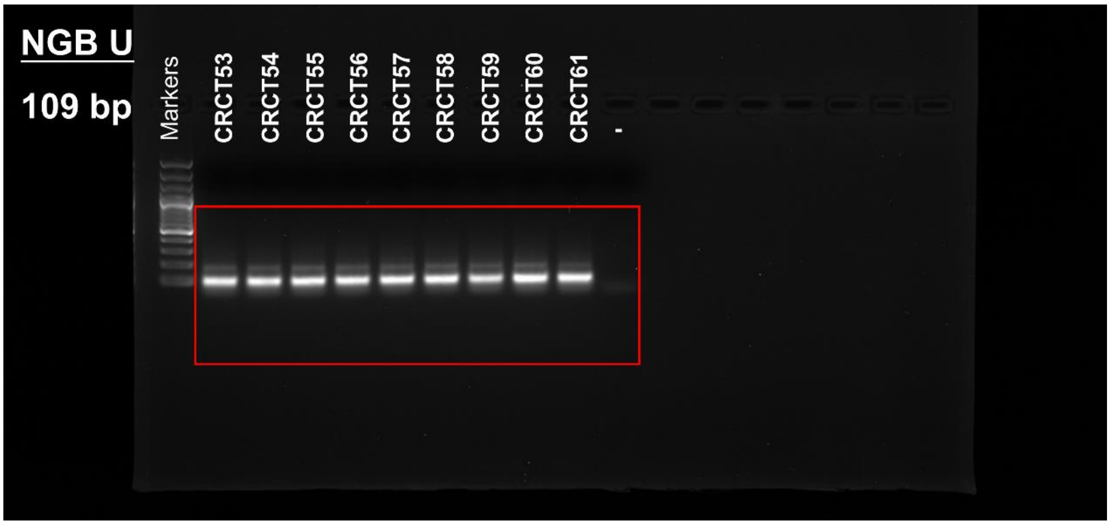

Figure 3A

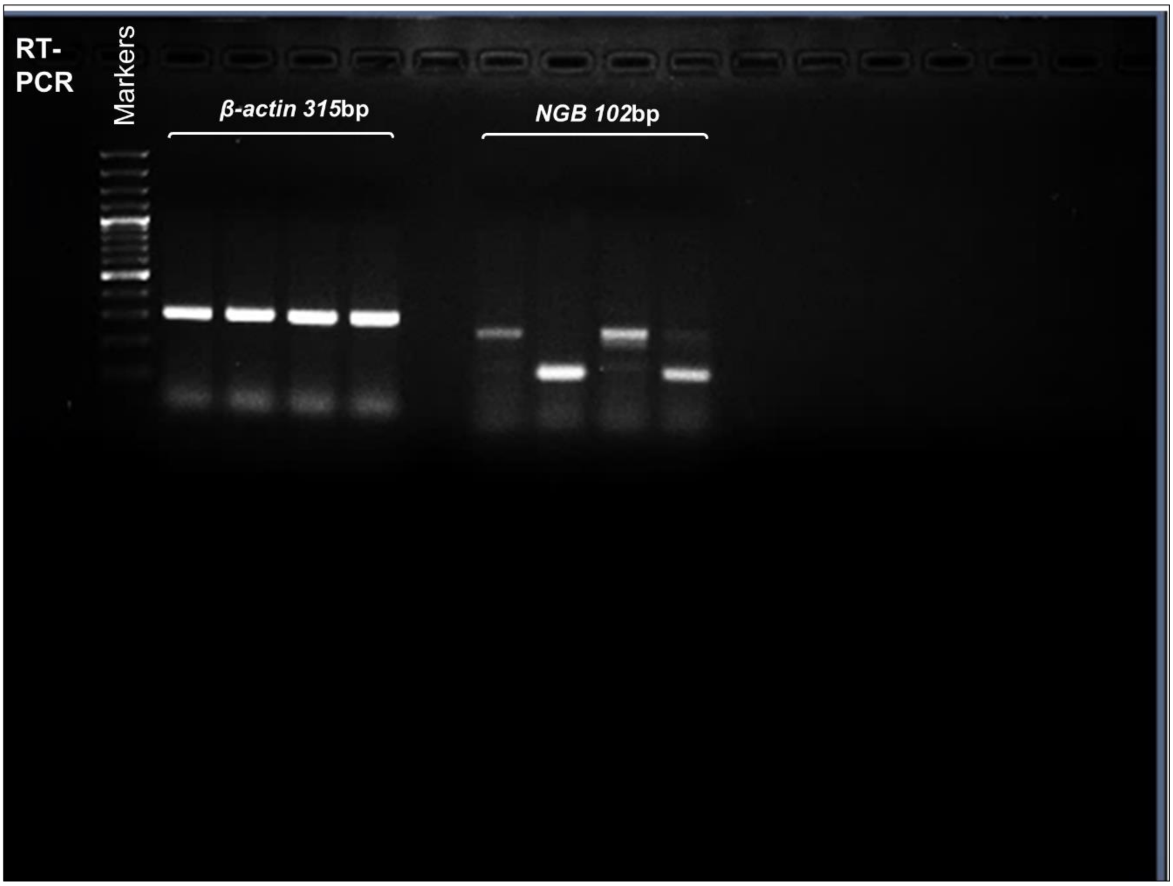

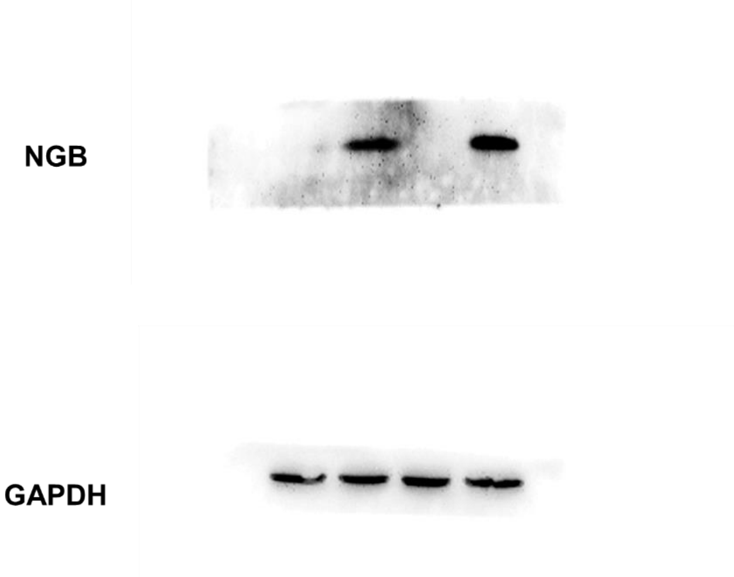

Figure 3F

Upper: HCT116, Bottom: Caco2

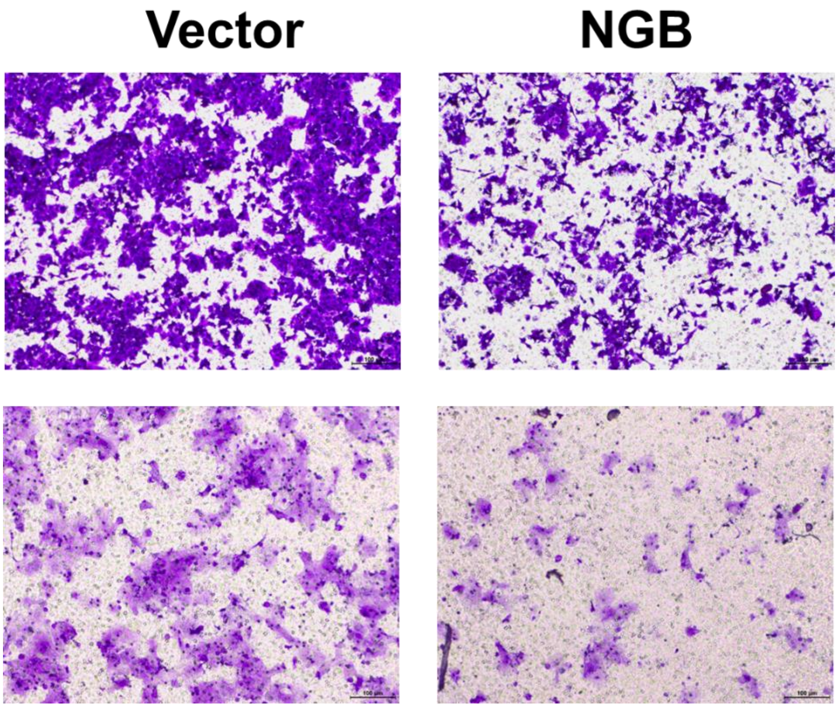

Figure 3G

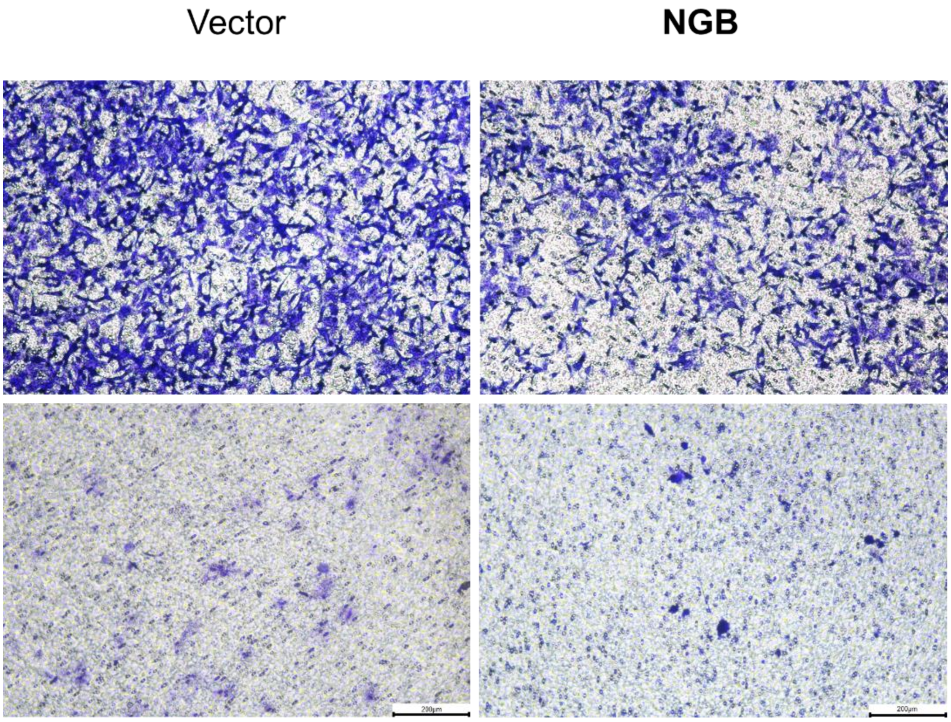

Figure 4B

Upper: HCT116 conditional medium,

Bottom: Caco2 conditional medium,

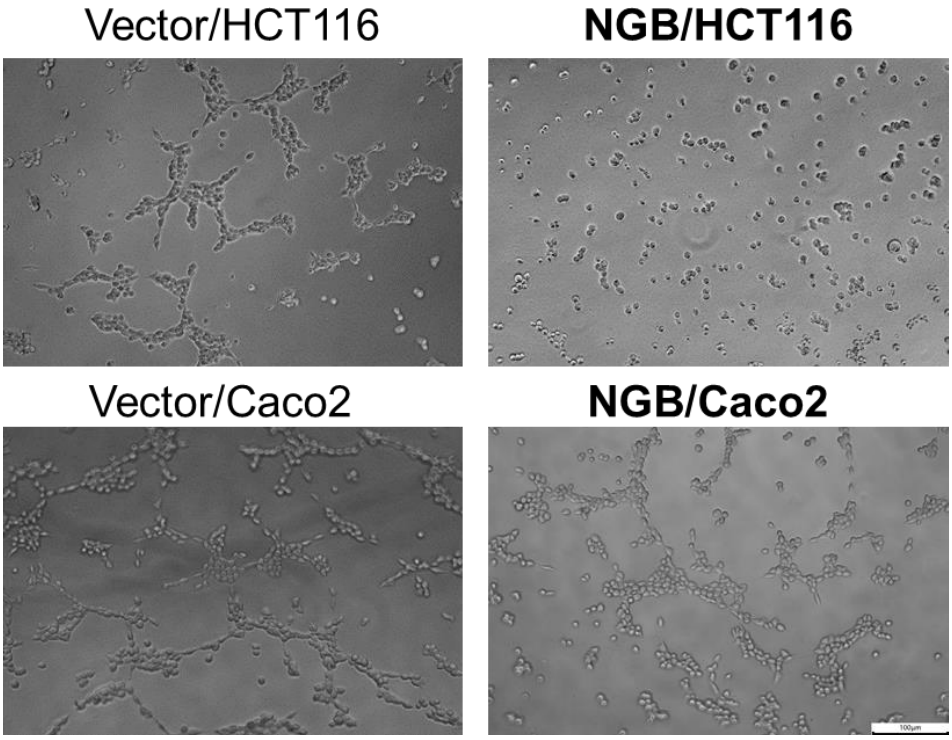

Figure 4C

Upper: HCT116, Bottom: Caco2

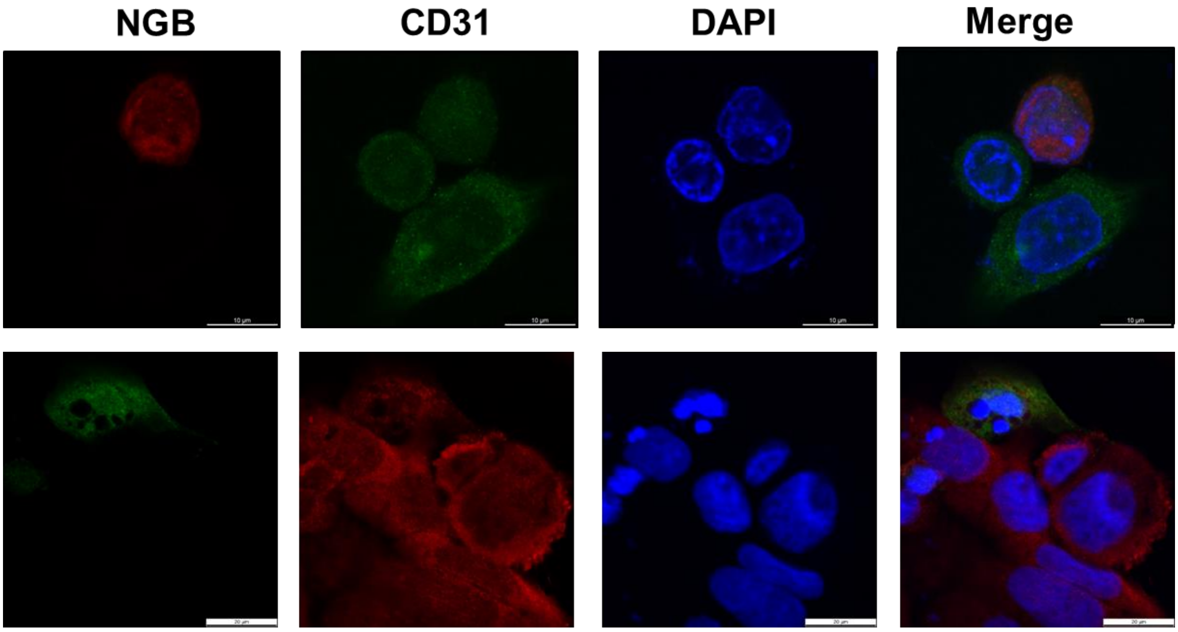

Figure 4D WB

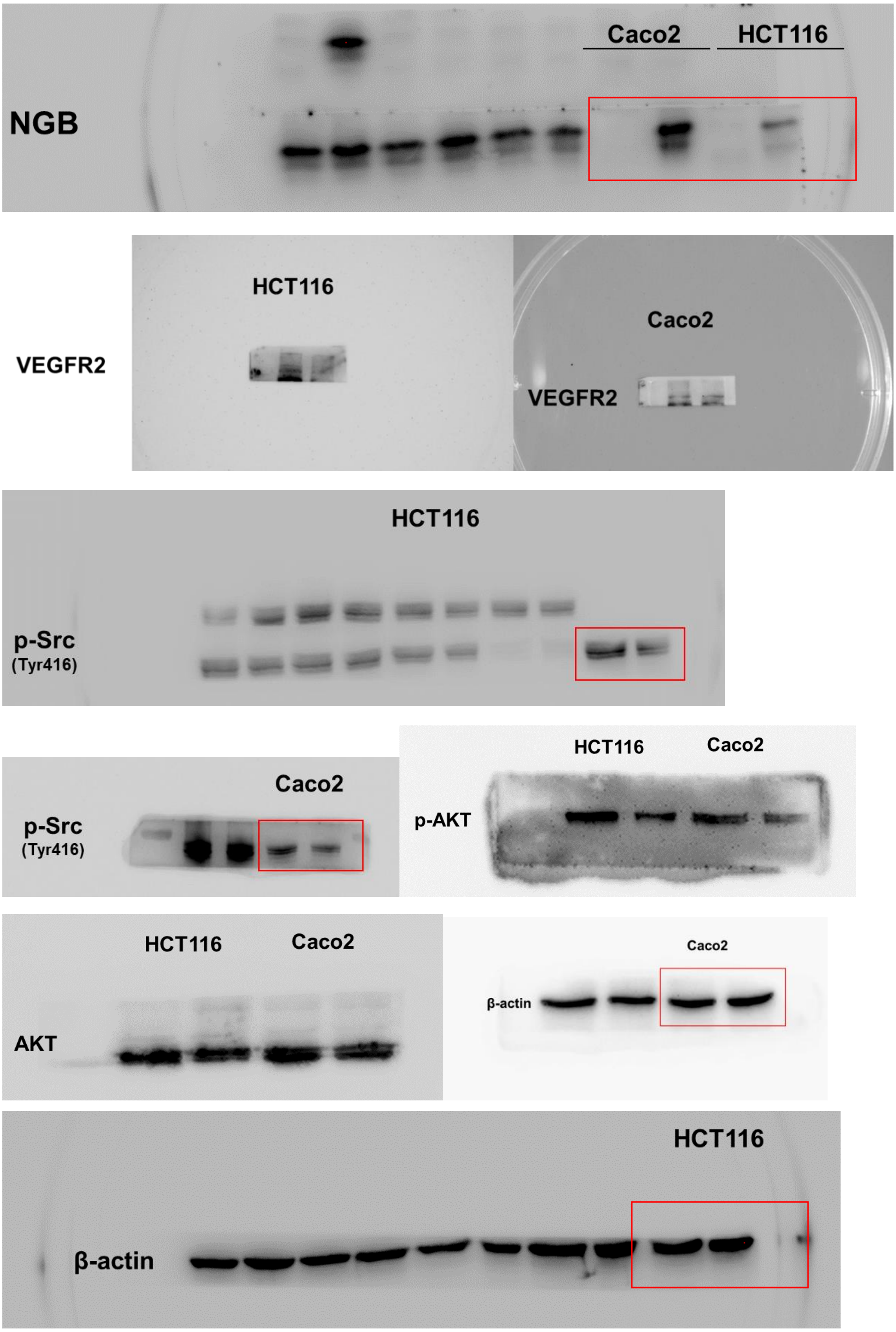

Caco2
HCT116
NGB
VEGFR2
HCT116
Caco2
VEGFR2
HCT116
p-Src
(Tyr416)
p-Src
(Tyr416)
Caco2
HCT116
Caco2
p-AKT
HCT116
Caco2
AKT
Caco2
β-actin
HCT116
β-actin

Figure 4D. WES

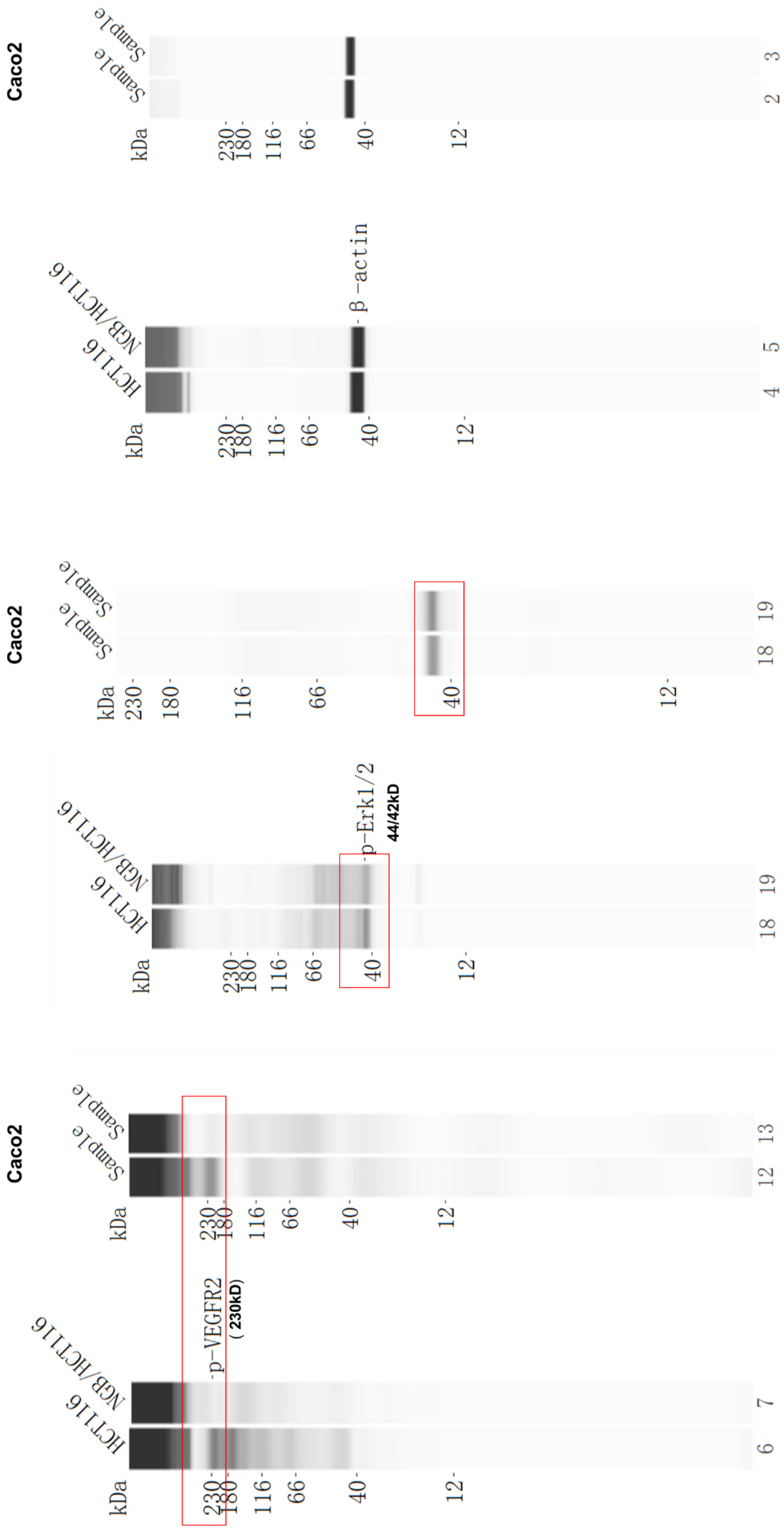

Figure 4E

Upper: HCT116, Bottom: Caco2

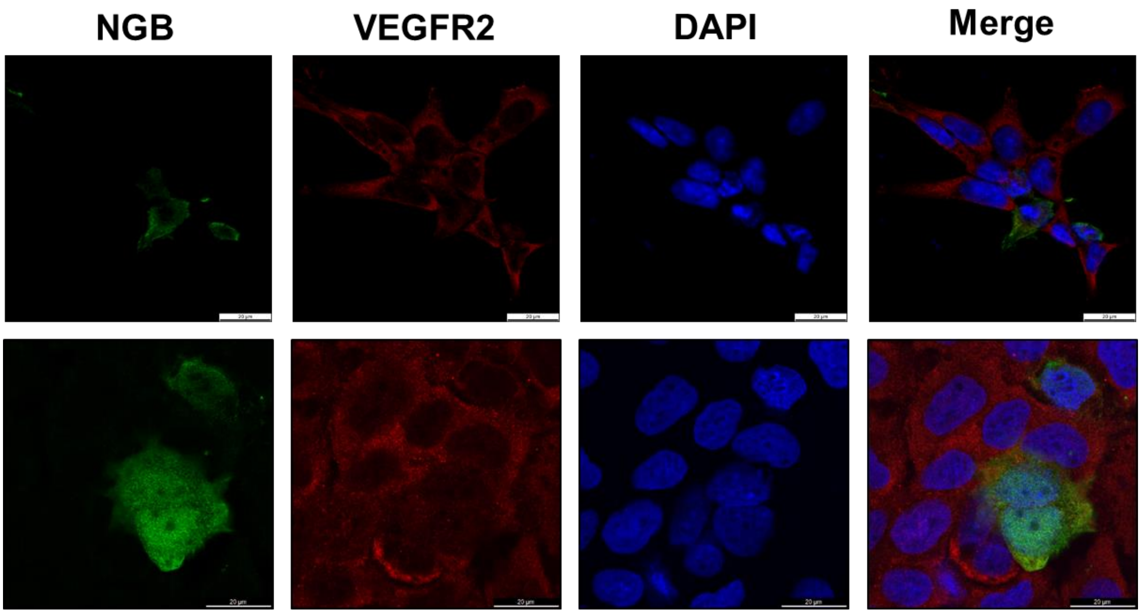

Figure 5B WB

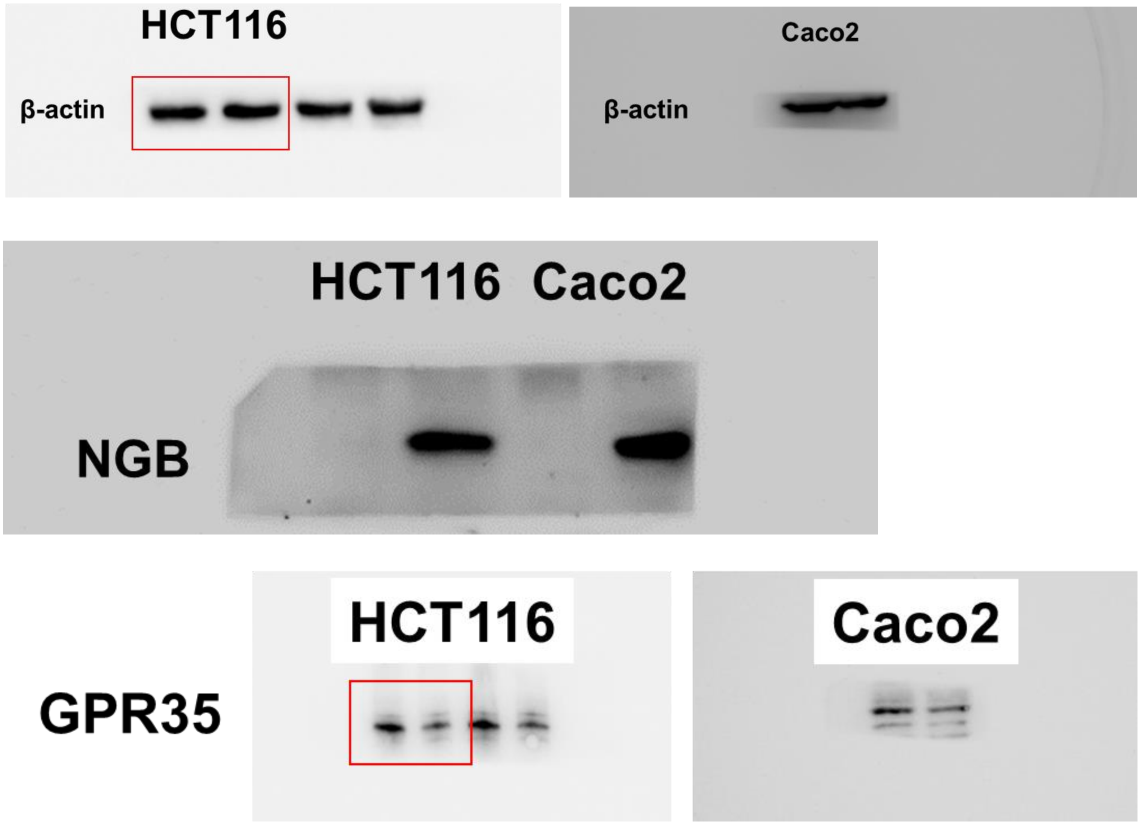

Figure 5C WB

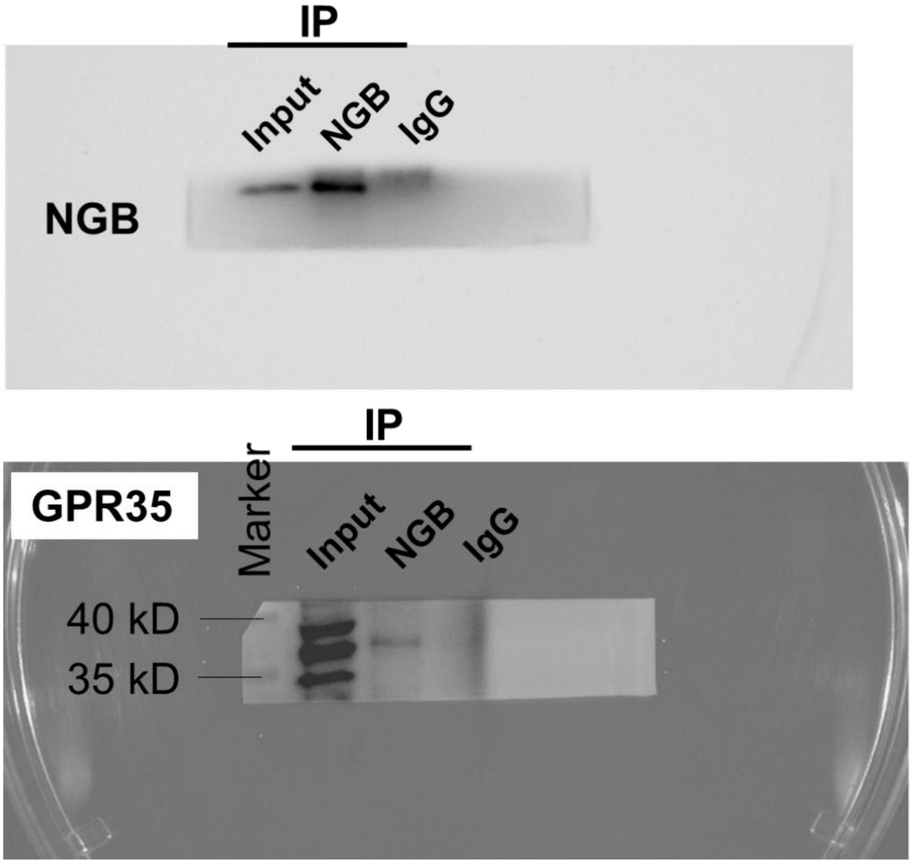

Figure 5D

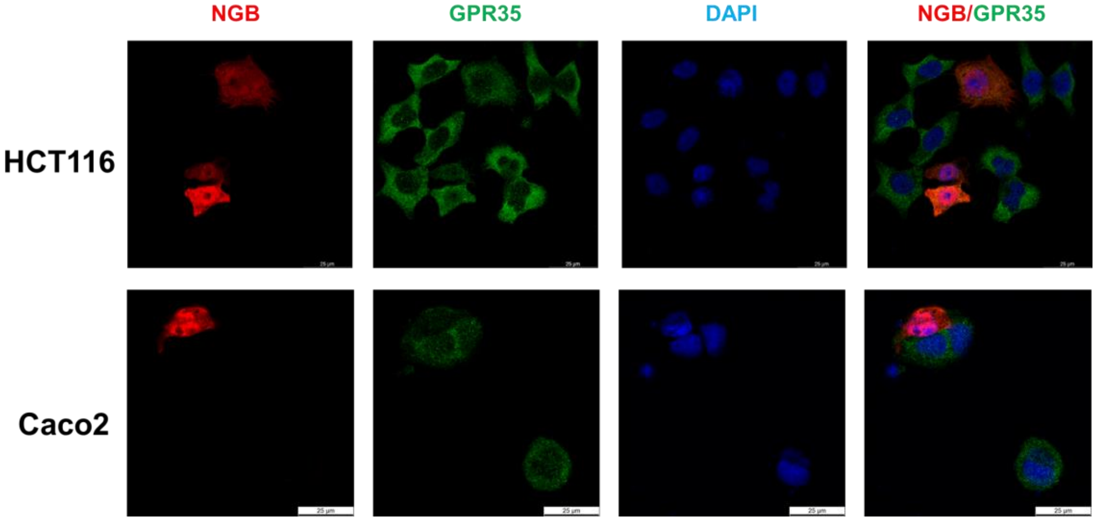

Figure 5E WB

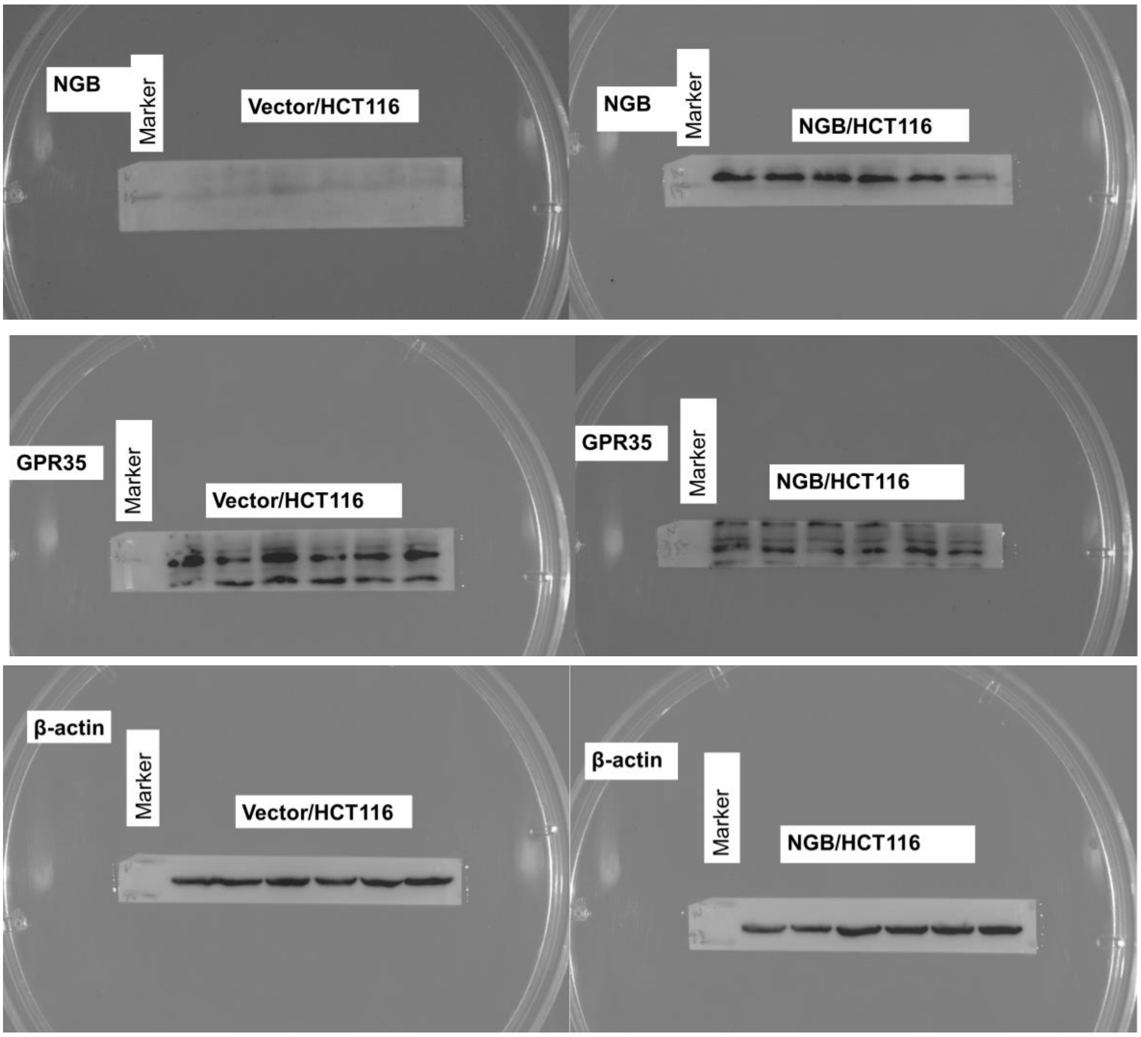
NGB
Marker
Vector/HCT116
NGB
Marker
NGB/HCT116
GPR35
Marker
Vector/HCT116
GPR35
Marker
NGB/HCT116
β-actin
Marker
Vector/HCT116
β-actin
Marker
NGB/HCT116

Figure 6B WB

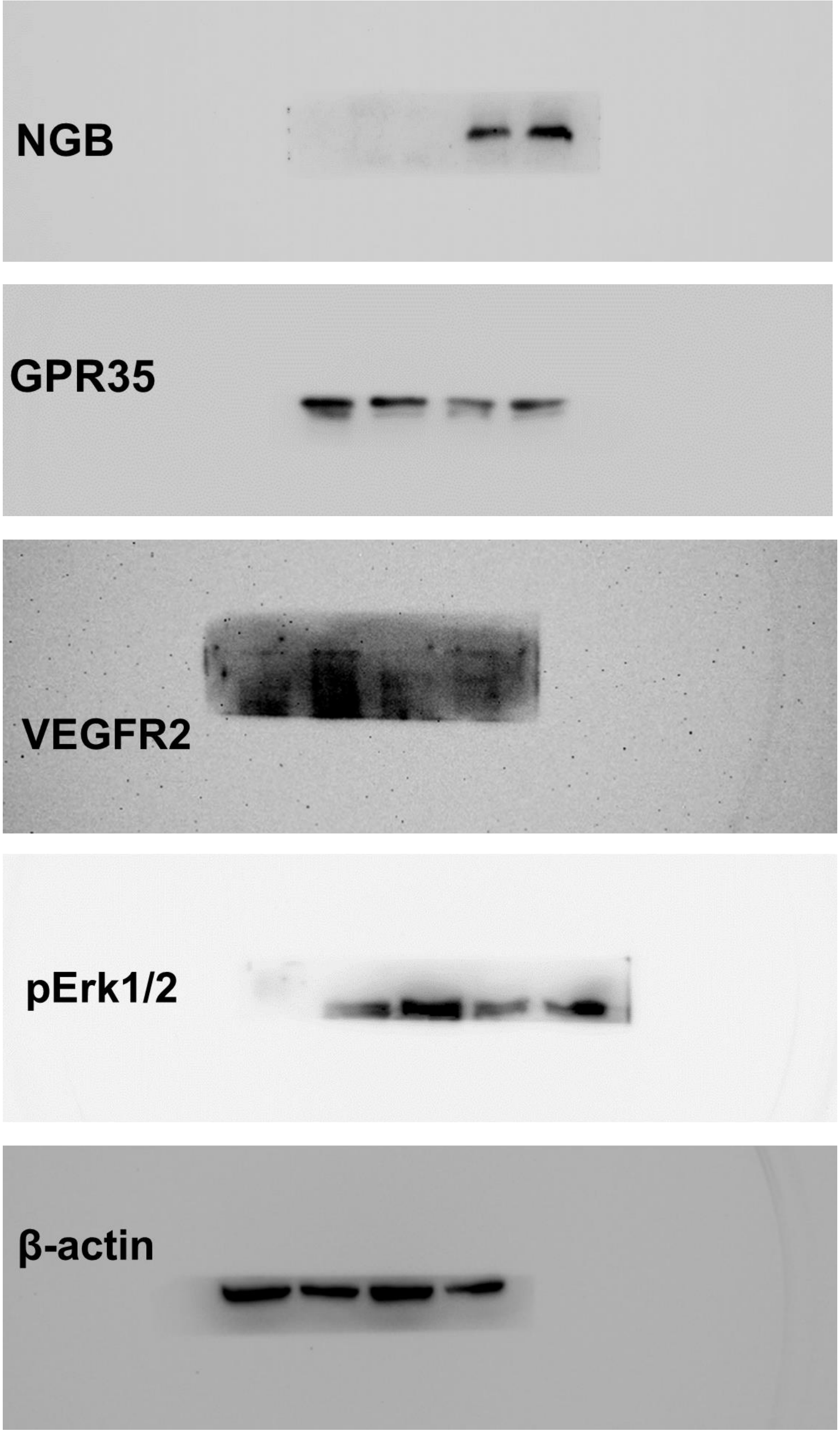

Figure 6C

HCT116 conditional medium,

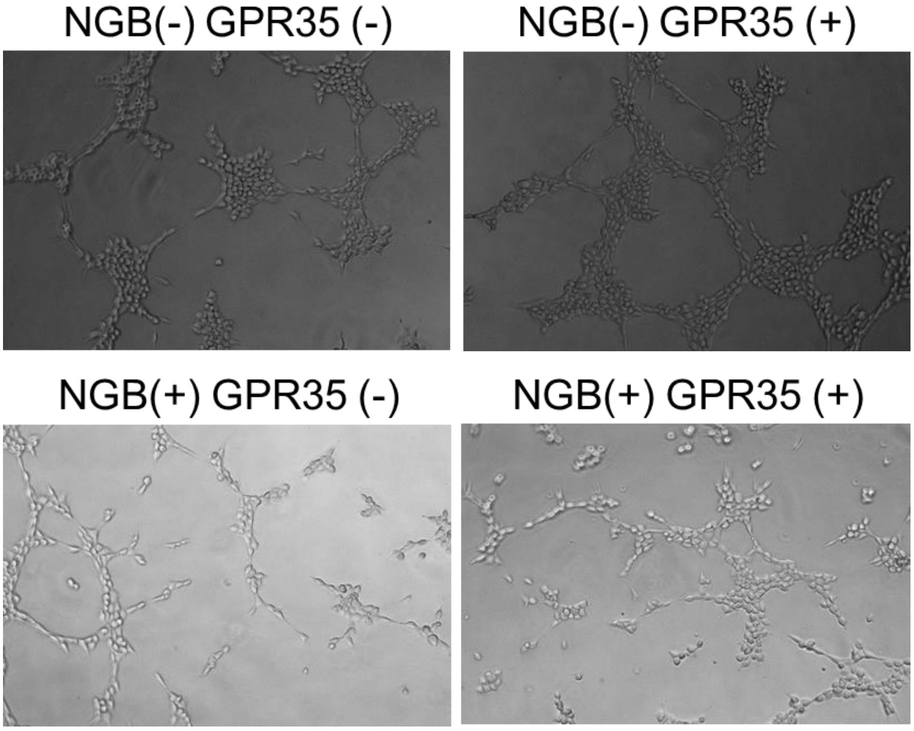

Caco2 conditional medium

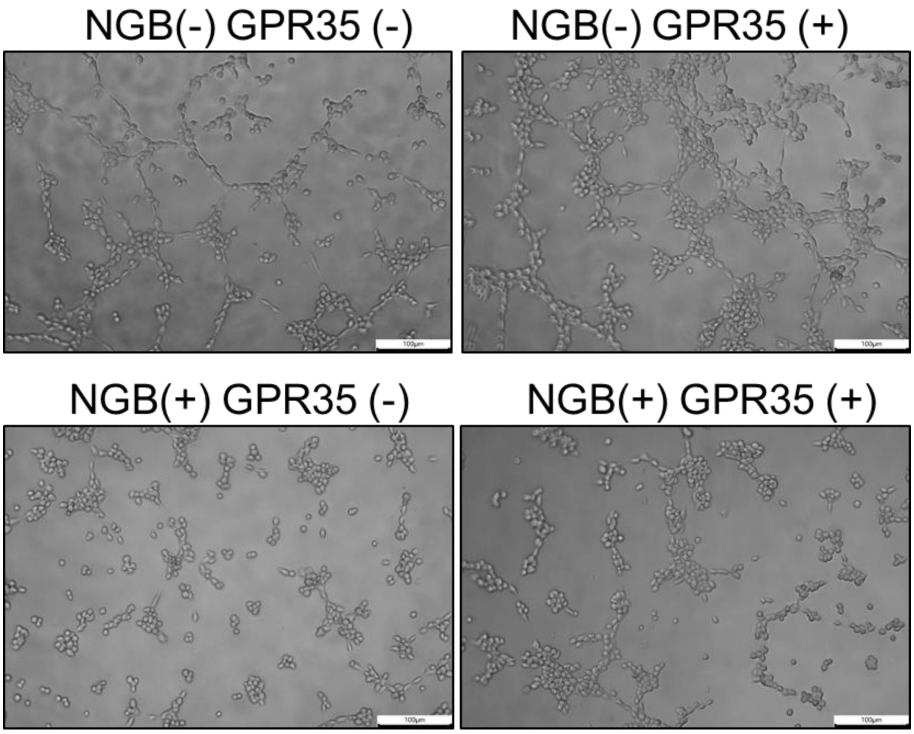

Figure 6D

HCT116-Invasion

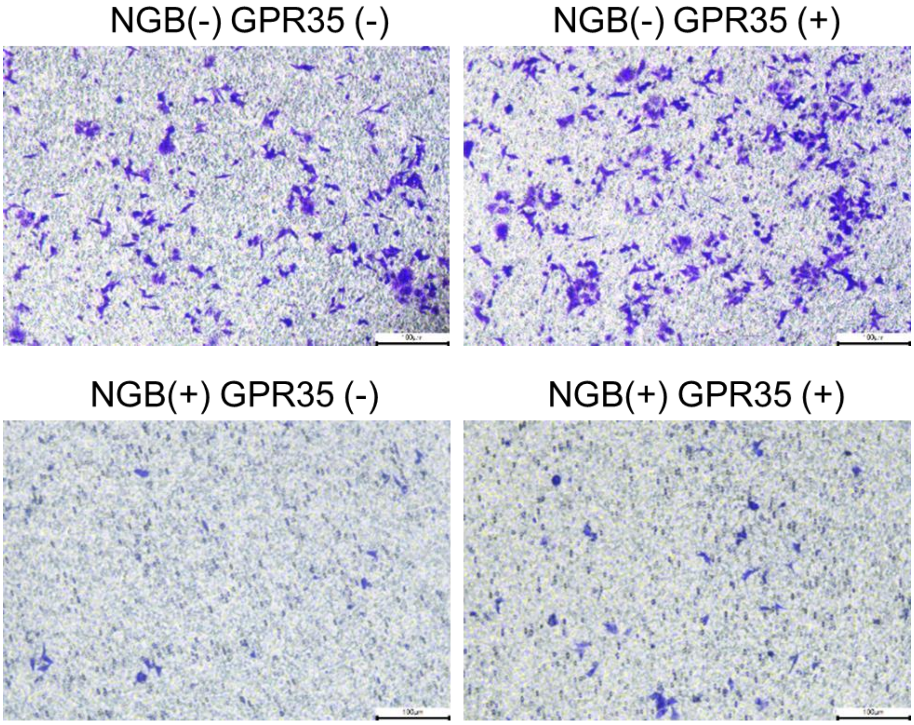

HCT116-Migration

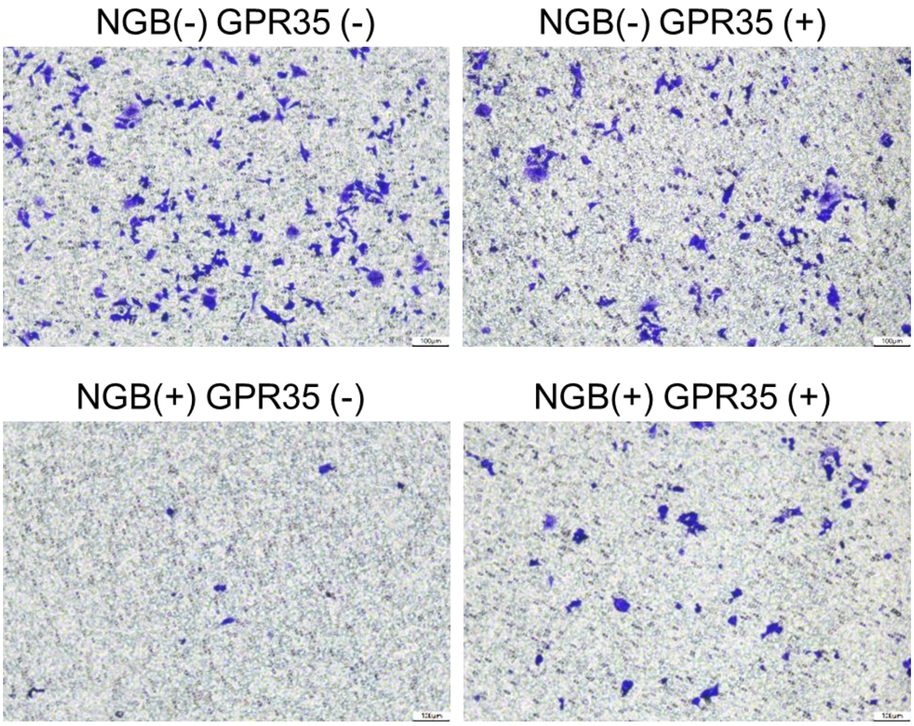

sFig 3D WB

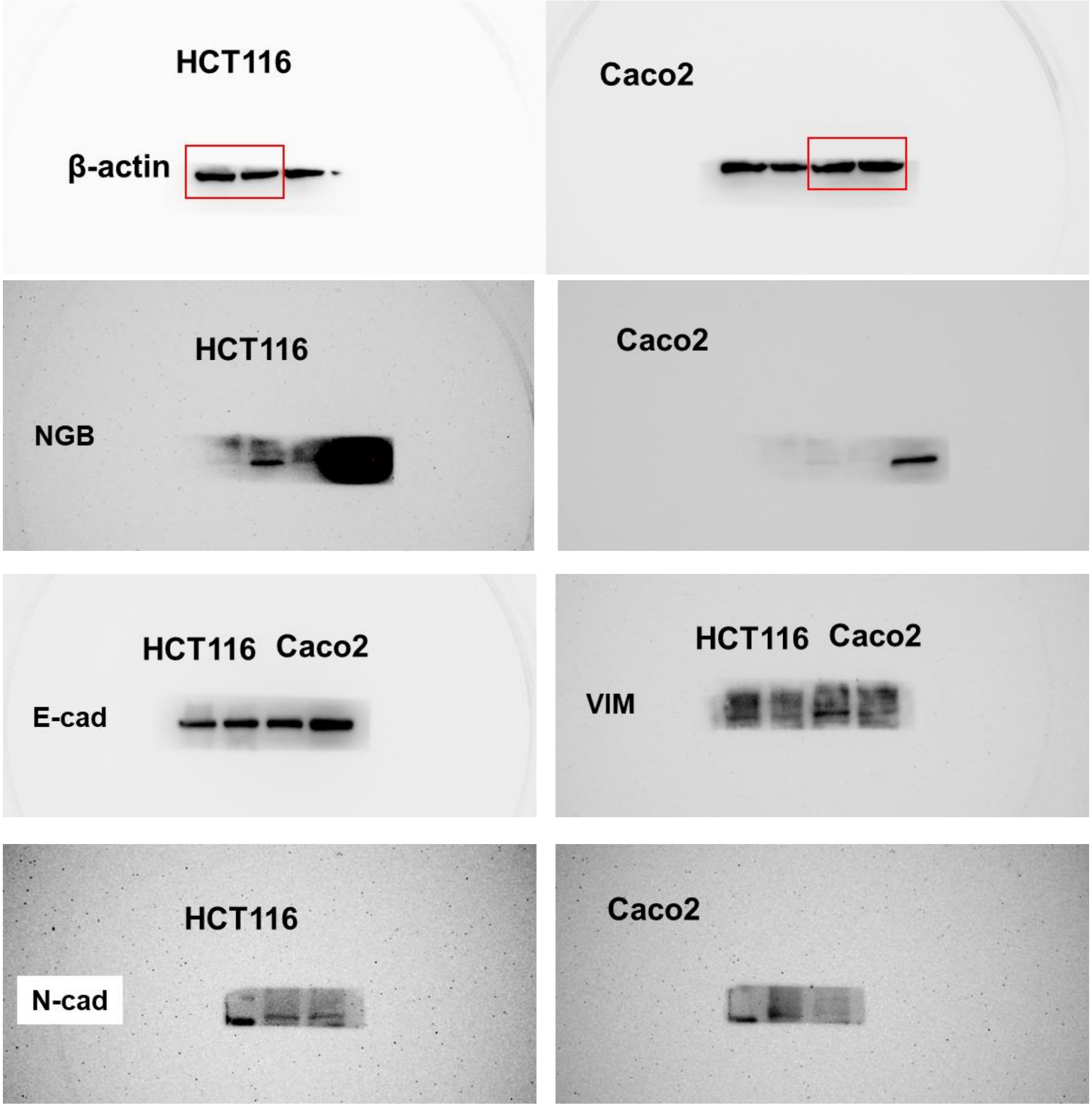
HCT116
β-actin
Caco2
HCT116
NGB
Caco2
HCT116 Caco2
E-cad
HCT116 Caco2
VIM
HCT116
N-cad
Caco2

sFigure 6B transwell

Upper: HCT116 conditional medium,

Bottom: Caco2 conditional medium

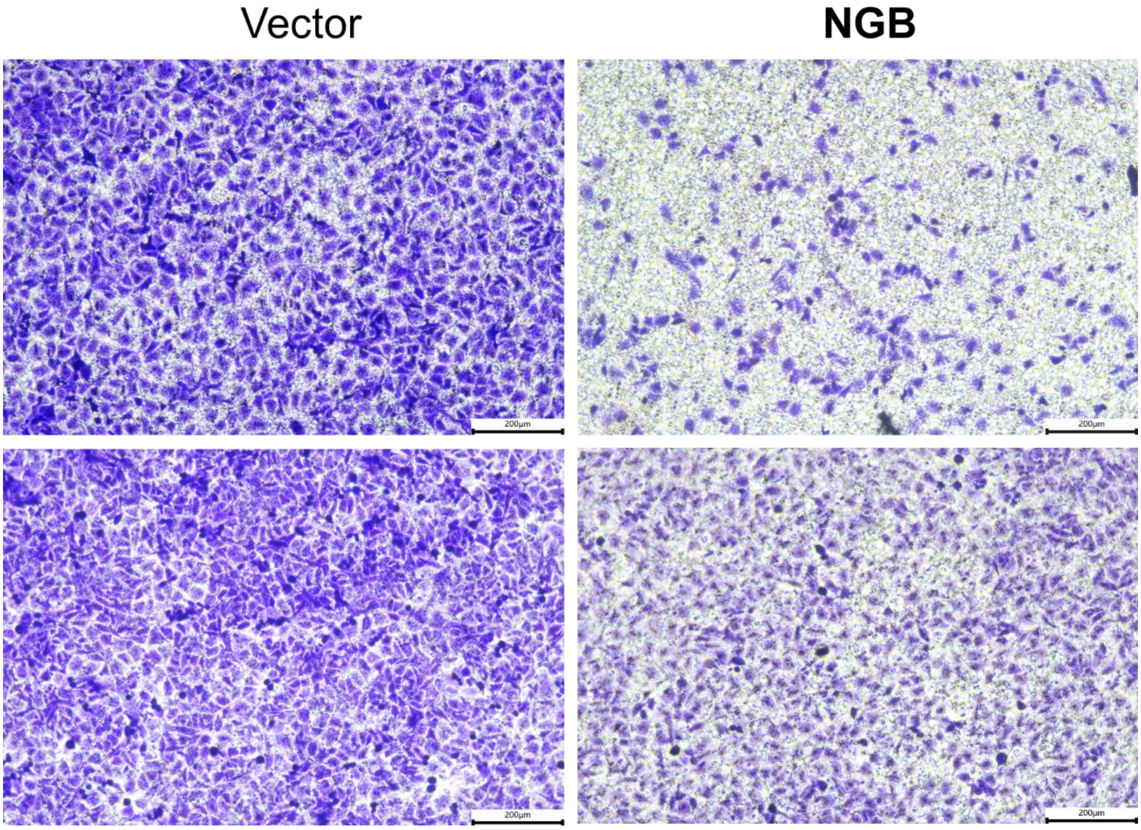

Supplement: Supplementary file 3 — Additional file 3. Original blots. [file 13148_2023_1472_MOESM3_ESM.pdf]
